# Small Extracellular Vesicle‐Derived Nidogen 1 Promotes HCC Carcinogenesis via Transglutaminase 3‐Mediated YAP/TAZ Signalling

**DOI:** 10.1002/jev2.70359

**Published:** 2026-08-17

**Authors:** Cherlie Lot Sum Yeung, Le Cui, Jingyi Wang, Ching‐Ping Chan, Tung Him Ng, Charlotte Jiaqi Lai, Xiaowen Mao, Sze Keong Tey, Michael Shing Yan Huen, Judy Wai Ping Yam

**Affiliations:** ^1^ Department of Pathology Li Ka Shing Faculty of Medicine, The University of Hong Kong Hong Kong China; ^2^ State Key Laboratory of Mechanism and Quality of Chinese Medicine Institute of Chinese Medical Sciences University of Macau Macao China; ^3^ Department of Surgery Li Ka Shing Faculty of Medicine, The University of Hong Kong Hong Kong China; ^4^ School of Biomedical Sciences Li Ka Shing Faculty of Medicine, The University of Hong Kong Hong Kong China; ^5^ State Key Laboratory of Liver Research The University of Hong Kong Hong Kong China; ^6^ Materials Innovation Institute for Life Sciences and Energy (MILES) The University of Hong Kong Shenzhen Institute of Research and Innovation (HKU‐SIRI) Shenzhen China

**Keywords:** hepatocellular carcinoma, intercellular communication, Nidogen 1, therapeutics, tumour microenvironment

## Abstract

Tumour‐derived small extracellular vesicles (sEV) are increasingly recognized as key mediators of cancer progression and metastasis by facilitating intercellular communication. In this study, we identify nidogen 1 (NID1), a basement membrane glycoprotein, as significantly enriched in sEV from metastatic hepatocellular carcinoma (HCC) cells. We demonstrate that the sEV‐mediated delivery of NID1 enhances oncogenic behaviours, including proliferation, invasion and metastasis. Knockout mice of NID1 exhibited a marked delay in liver tumour formation induced by N‐diethylnitrosamine (DEN) and carbon tetrachloride (CCL4), or hydrodynamic injection delivery of oncogenes, underscoring the role of NID1 in hepatocarcinogenesis. Importantly, we find that a stiffer extracellular matrix, a characteristic of tumour microenvironments, stimulates increased NID1 level in sEV. Using NID1 mutants, we identify the C‐terminal domain as critical for its oncogenic functions, including promoting tumour growth and metastasis. Proteomic profiling reveals that transglutaminase 3 (TGM3) interacts with NID1 via its C‐terminus. Subsequent analyses indicate that sEV‐NID1 elevates TGM3 levels, leading to dysregulation of integrins and activation of the YAP/TAZ signalling pathway, thereby contributing to tumour progression. To explore therapeutic potential, we developed a novel monoclonal anti‐NID1 antibody (anti‐NID1 mAb) and evaluated its efficacy in multiple HCC mouse models. The antibody markedly suppressed liver tumour growth and metastasis of human and murine HCC cells. The therapeutic effect was further enhanced when combined with sorafenib, a standard treatment for HCC. Our findings delineate a novel sEV‐NID1‐TGM3 regulatory axis that promotes hepatocarcinogenesis and metastasis and suggest that targeting sEV‐delivered NID1 with our homemade monoclonal antibody offers a promising avenue for therapeutic intervention in HCC.

## Graphical abstract

NID1‐rich sEVs released by HCC cells are internalized by recipient cells, where the C‐terminal region of NID1 binds to and stabilizes TGM3. This activates integrin‐FAK/Src/AKT and Hippo‐YAP/TAZ signalling, thereby promoting HCC proliferation, colony formation, migration, invasion, tumour growth, and lung metastasis.

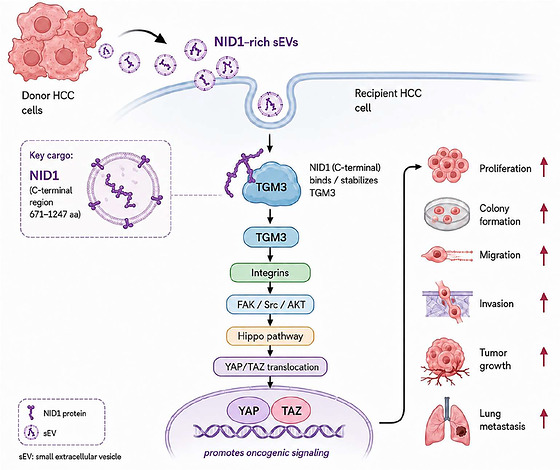

## 1 Introduction

Hepatocellular carcinoma (HCC) accounts for more than 80% of liver cancer cases (Bosch et al. 2004), with a 5‐year survival rate as low as 20% (Calderon‐Martinez et al. 2023). The high lethality of HCC can be attributed to its asymptomatic nature, as the majority of patients were only diagnosed at a late, advanced stage. In HCC, pre‐existing cirrhosis is a major risk factor, which refers to a nonreversible stage of fibrous thickening (Fattovich et al. 2004), leading to increasing crosslinking and contraction of the extracellular matrix (ECM) (Ishihara and Haga 2022; Puig et al. 2015). Liver fibrosis usually arises from continuous hepatocyte injuries that are triggered by hepatotoxic substances, viral infections or metabolic disorders (Koyama and Brenner 2017). Under such conditions, inflammatory cells are recruited and the mechanism of repair and replace are activated. Subsequently, scar tissues and ECM components are deposited to replace the injured parenchyma (Neubauer et al. 2001). When fibrosis continues, cellular tension is enhanced and leads to ECM remodelling and the activation of multiple signalling pathways, such as Wnt and Notch (Miao et al. 2013; Ni et al. 2018). These studies highlighted the dynamic interaction of ECM components, sEV, and oncogenic signalling within the tumour microenvironments.

Small extracellular vesicles (sEV) are secreted by almost all cells and are important mediators for intercellular communication (Meldolesi 2018). Despite their rather small size of around 30–150 nm, their lipid bilayer membrane allows a diverse number of biomolecules, such as proteins, lipids and nucleic acids, to be stably enclosed and transported to recipient cells (Gurung et al. 2021; Lane et al. 2015). Unlike other vesicles and nanoparticles, sEV are formed by inward budding of the plasma membrane into intraluminal vesicles and are released by fusion of multivesicular bodies with the membrane (Han et al. 2022). Upon secretion, sEV can be delivered to targeted cells based on their surface markers and induce various signals via receptor‐ligand interaction or endocytosis in recipient cells (Sadeghi et al. 2023). Apart from normal cells, tumour cells were also shown to hijack sEV for transferring oncogenic materials to support their growth and expansion (Whiteside 2016). Furthermore, it has been reported that ECM stiffening promoted sEV secretion from tumour cells via AKT and subsequent GTP‐loading to Rab8 (Wu et al. 2023). In our previous study, we have shown that sEV‐derived nidogen 1 (sEV‐NID1) played a crucial role in promoting extrahepatic metastasis by activating pulmonary fibroblasts to secrete tumour necrosis factor 1 (Mao et al. 2020). Treatment of cellular NID1 with a homemade monoclonal antibody was further demonstrated to bring promising results in various cancers (Xue et al. 2025). However, what triggers NID1's enrichment in sEV and their downstream activator remains largely unknown.

Being a major component of the basement membrane (BM), NID1/enactin serves as a linker glycoprotein essential for the organization of intricate BM ternary structures (Vasudevan et al. 2010). NID1 is comprised of 3 globular domains, G1, G2 and G3, each contains binding sites for different ECM components (Zhou et al. 2022). Dysregulation of NID1 level has been shown to cause irregular BM morphogenesis and the activation of integrin‐mediated signalling (Töpfer and Holz 2024). Aberrant NID1 expression has also been associated with mixed results in carcinogenesis. For instance, high NID1 level was associated with aggressiveness, stemness and chemoresistance in ovarian, breast and endometrial cancer (Pedrola et al. 2015; Urooj et al. 2020; Zhou et al. 2017). In HCC, NID1‐enriched vesicles have been shown to facilitate extrahepatic metastasis, and the inhibition of NID1 by transmembrane serine protease 2 hampers the migration and invasion ability of cancer cells (Ma et al. 2023). Interestingly, in certain cancers, NID1 functions as a tumour suppressor. For example, full length NID1 promoted a M1‐like macrophage polarization via integrin αvβ3 receptor binding, leading to antitumorigenic actions in melanoma and mammary tumour (Caracuel‐Peramos et al. 2025). In triple negative breast cancer, NID1 was found to be downregulated and could suppress HIF‐1⍺ downstream transcription (Lee et al. 2025). In HCC, both TCGA dataset and Kaplan–Meier (KM) survival analysis showed an upregulation of NID1 expression (Xue et al. 2025). However, it remains largely unclear what downstream activator or signalling pathway are involved.

In this study, we delineate the role of tumour‐derived sEV‐NID1 in HCC oncogenesis and address the contribution of stiff‐matrix in promoting NID1's enrichment in sEV. By establishing NID1 mutants and performing coimmunoprecipitation‐coupled mass spectrometry (co‐IP‐MS), we identified the C terminus domain to be critical for sEV‐NID1's oncogenic activities and transglutaminase 3 as a key binding partner. sEV‐NID1 mediated induction of TGM3 is found to cause aberrant integrin expression and the activation of the YAP/TAZ signalling pathway. Finally, we demonstrate that targeting NID1 by homemade monoclonal antibody (anti‐NID1 mAb) suppresses tumorigenesis and metastasis, suggesting a potential therapeutic strategy.

## 2 Results

### 2.1 sEV‐Mediated NID1 Delivery Contributes to HCC Carcinogenesis

**FIGURE 1:**
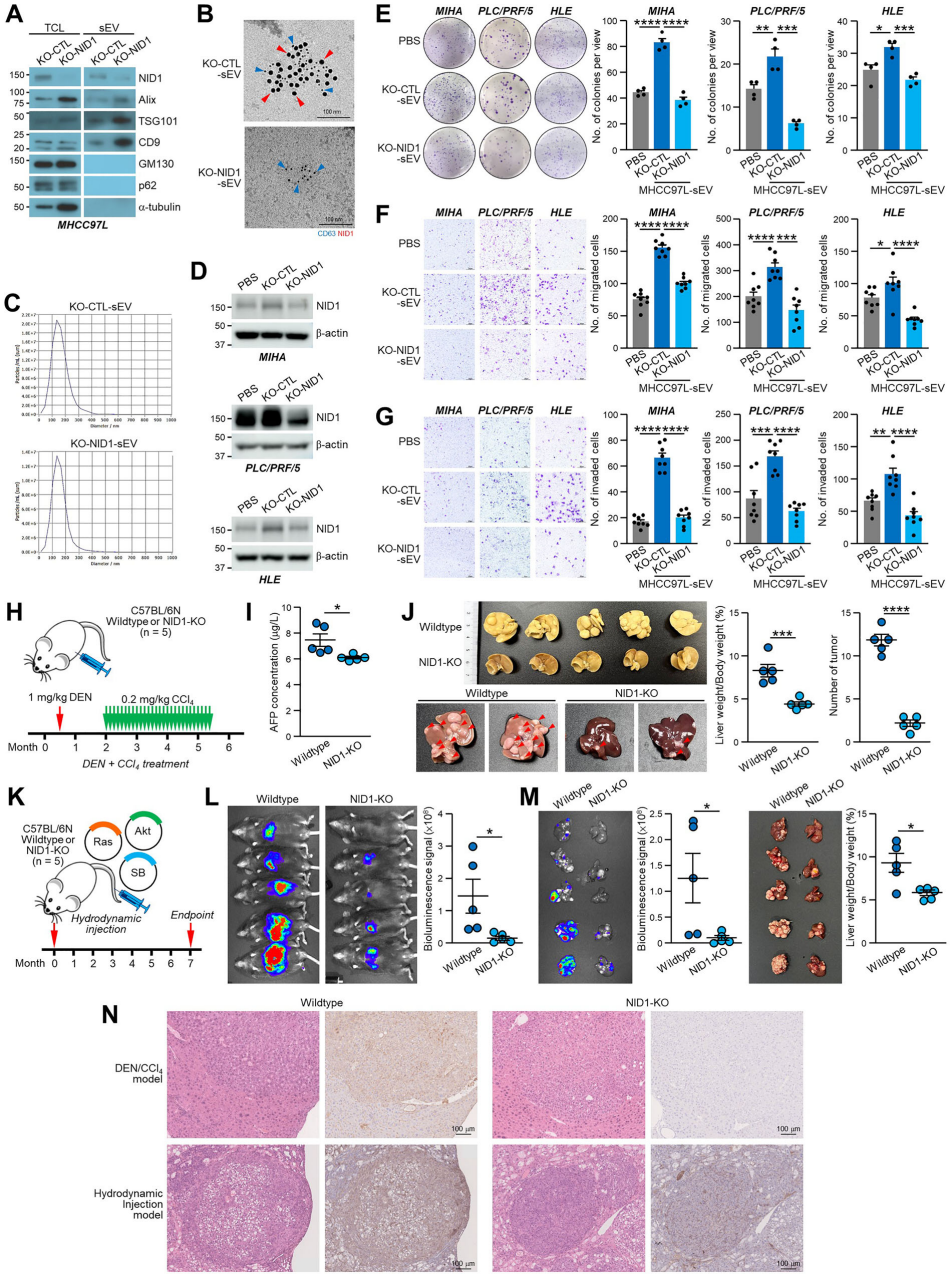
sEV‐mediated NID1 delivery contributes to HCC carcinogenesis (A) Immunoblotting of NID1, positive exosome markers (Alix, TSG101 and CD9) and negative exosome markers (GM130, p62, ⍺‐tubulin) in total cell lysate and sEV of MHCC97L KO‐CTL and KO‐NID1 cells. (B) Representative electron micrographs of sEV subjected to immunogold labelling using anti‐CD63 (blue) and anti‐NID1 (red), coupled to 5‐ and 15‐nm gold particles, respectively. Scale bar, 100 nm. (C) The size distribution of sEV derived from MHCC97L KO‐CTL and KO‐NID1 were measured by ZetaView nanoparticle tracking analyser. (D) Immunoblotting of NID1 expression in MIHA, PLC/PRF/5 and HLE cells upon treatment by PBS, MHCC97L KO‐CTL‐ and KO‐NID1‐derived sEV. (E–G) MIHA, PLC/PRF/5 and HLE cells were treated with PBS, MHCC97L KO‐CTL‐ and KO‐NID1‐sEV. Representative images and numbers of colonies (E) migrated (F) and invaded (G) cells were shown and quantified. (H) A schematic diagram of chemical‐induced HCC mouse model using DEN (1 mg/kg) and CCl_4_ (0.2 mg/kg). (I) Serum AFP level of widetype and NID1‐KO mice were analysed. (J) Images of excised livers and tumours (red arrow) were shown. Liver/Body weight ratio and number of tumours were quantified. (K) A schematic diagram of hydrodynamic tail vein injection model. (L–M) Bioluminescence signals of whole mouse (L) and excised liver tissues (M) were shown and quantified. (N) Representative H&E‐stained and immunohistochemical staining of NID1 in liver tumour and surrounding tissue were shown. Scale bar, 100 µm.

Our previous studies using shRNA demonstrated that sEV‐NID1 is required for HCC oncogenesis and metastasis (Mao et al. 2020). To consolidate the findings, we further employed CRISPR/Cas9 technology to create stable NID1‐knockout (KO‐NID1) in metastatic MHCC97L cells. As compared to vehicle control (KO‐CTL), depletion of NID1 expression was observed in KO‐NID1 cells (Figure 1A). We then isolated sEV from the conditioned medium of KO‐CTL and KO‐NID1 cells, which verified reduction in NID1 expression on sEV using both immunoblotting and transmission electron microscopy (Figure 1A,B). The purity, integrity and size of sEV collected were also validated (Figure 1A–C). We further analysed the functional effect of sEV‐NID1 on a normal human hepatocyte (MIHA) and two non‐metastatic HCC cells (PLC/PRF/5 and HLE). Recipient cells treated with KO‐CTL‐sEV showed a marked elevation in cellular NID1 level, while those treated with PBS and KO‐NID1‐sEV did not (Figure 1D). A lower level of NID1 showed hampered sEV‐mediated promotive effect on colony formation, migration and invasion of recipient cells (Figure 1E–G). The importance of NID1 in HCC carcinogenesis *in vivo* was further assessed using a genetic engineered NID1‐KO mouse model. Two clinically relevant HCC models were then established on wildtype (WT) and NID1‐KO mouse strains. In the chemical‐induced model using diethylnitrosamine (DEN) and carbon tetrachloride (CCl_4_) model, level of alpha‐fetoprotein (AFP) tumour marker and number of tumour nodules were significantly reduced in NID1‐KO mice (Figure 1H–J). Likewise, using the hydrodynamic tail vein injection (HDTVi) of proto‐oncogenes (N‐RasV12 and Myr‐AKT) model, hepatocarcinogenesis was also significantly inhibited when NID1 was depleted (Figure 1K–M). In addition, histological and immunohistochemical analyses of liver sections showed that NID1 expression was enhanced in tumour regions in the WT group, but such elevation was not observed in NID1‐KO mice (Figure 1N). Taken together, these data demonstrate that sEV‐mediated NID1 delivery is associated with tumour aggressiveness in HCC.

### 2.2 Elevated sEV‐NID1 is Related to Matrix Stiffening

**FIGURE 2:**
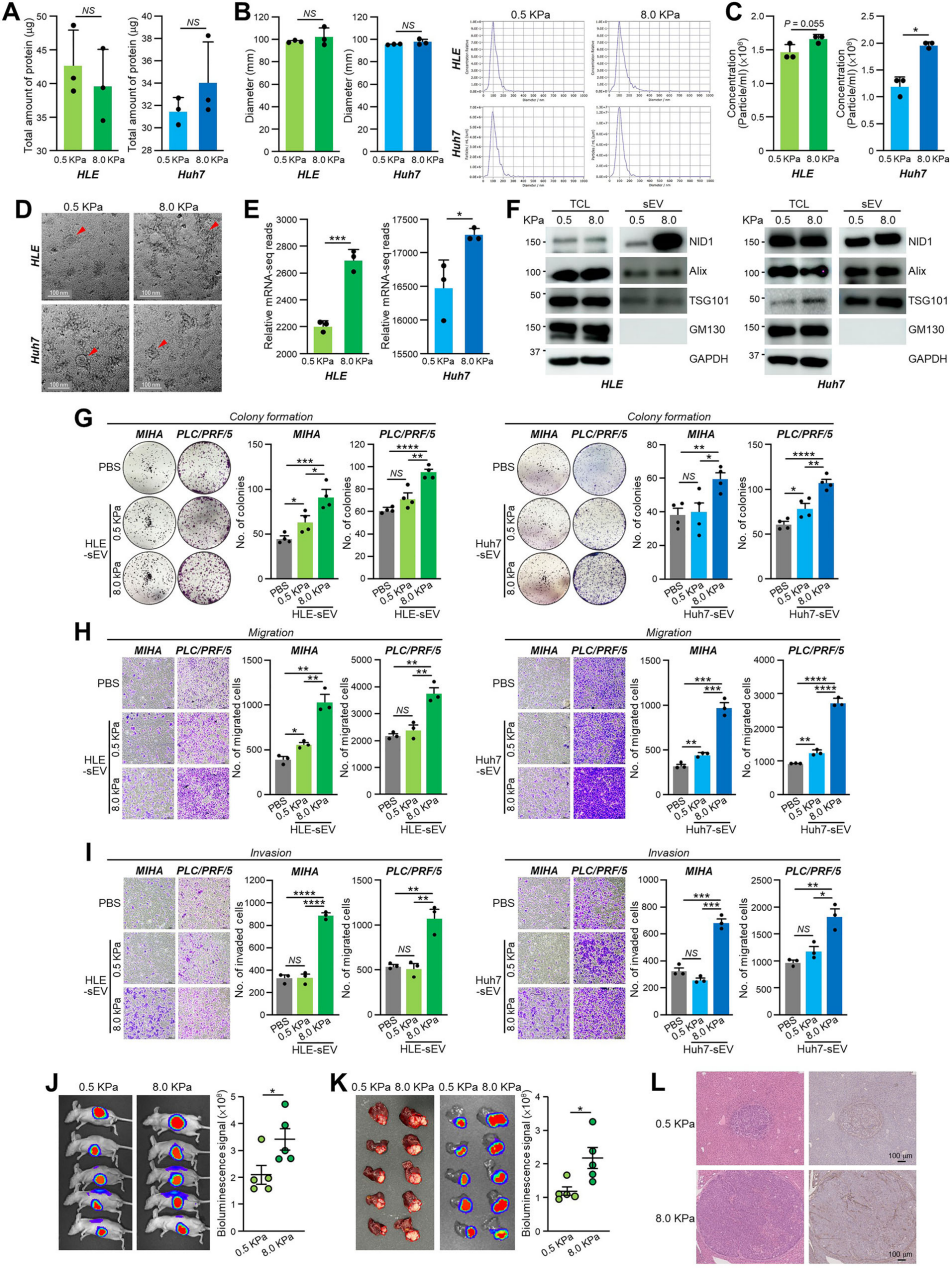
Elevated sEV‐NID1 is related to matrix stiffening (A–C) Total amount of protein (µg) (A), size distribution (B)and particle concentration (C) were quantified in sEV isolated from HLE and Huh7 cells cultured in 0.5 and 8 kPa stiffness matrix. (D) Representative electron micrographs of sEV isolated from HLE and Huh7 cells cultured in 0.5 and 8 kPa stiffness matrix were subjected to negative staining. (E) Relative mRNA sequencing reads of HLE and Huh7 cells cultured in different stiffness were quantified. (F) Immunoblotting of NID1, positive sEV markers (Alix and TSG101) and negative sEV markers (GM130) in total cell lysate and sEV of HLE and Huh7 cells cultured in different stiffness. Equal amounts of total sEV protein were loaded. (G–I) MIHA and PLC/PRF/5 cells were subjected to treatment by PBS, 0.5 kPa‐sEV and 8 kPa‐sEV derived from HLE (*left*) or Huh7 (*right*) cells, respectively. Representative images and numbers of colonies (G) migrated (H) and invaded (I) cells were shown and quantified. (J–K) PLC/PRF/5 cells were cotreated with sEV isolated from HLE cells cultured on 0.5 and 8 kPa stiffness prior to orthotopic liver implantation. Bioluminescence signal of whole mouse (J) and excised liver and tumours (K) were shown and quantified. (L) Representative H&E‐stained and immunohistochemical staining of NID1 in liver tumour and surrounding tissue of mice orthotopically injected with PLC/PRF/5 cells cotreated with different stiffness sEV. Scale bar, 100 µm.

Studies have demonstrated that cells cultured on a stiffer matrix would secrete small extracellular vesicles (exosomes) with higher protein level (Wu et al. 2023; Xiao et al. 2022). To investigate whether matrix stiffness would affect NID1's level in sEV, we cultured two non‐metastatic HCC cell lines (HLE and Huh7) on soft (0.5 kPa) and stiff (8 kPa) rigidity plates. From our isolated sEV, our data showed no significant difference in total amount of protein, as measured by Bradford protein assay, between sEV from cells cultured on different stiffness (Figure 2A). The diameter and morphology of sEV, as determined by nanoparticle tracking analysis and transmission electron microscopy, also showed no obvious changes (Figure 2B and D). However, cells cultured on a stiffer matrix had an elevated concentration of sEV (particle/ml) (Figure 2C). Subsequently, we explored whether sEV‐NID1 level was affected. Interestingly, our RNA sequencing data revealed that cells cultured on stiffer matrix exhibited higher level of NID1 transcripts compared to cells cultured on a soft matrix (Figure 2E). However, further analysis showed that NID1 cellular level was not affected, but instead sEV‐NID1 protein level was elevated (Figure 2F and Table S1). Suppression of Alix or TSG101 in cells cultured on a stiffer matrix was found to reduce NID1 levels in sEV, without affecting cellular NID1 expression, suggesting that the sorting of NID1 into sEV is dependent on Alix and TSG101 (Figure S1). Given the high sEV‐NID1 level secreted by cells cultured on stiff matrix, we further examined their functional role in tumour progression. Treatment of stiff‐sEV promoted the colony formation, migration and invasion of normal hepatocyte (MIHA) and non‐metastatic HCC cell (PLC/PRF/5) (Figure 2G–I). To investigate the impact of stiff matrix‐induced sEV‐NID1 *in vivo*, PLC/PRF/5 cells co‐treated with different stiffness sEV were orthotopically injected to nude mice liver. Mouse group co‐treated with 8 kPa‐sEV exhibited a more aggressive tumour growth and also higher NID1 expression (Figure 2J–L). These results suggest that liver stiffness is responsible for sEV‐NID1 high expression in HCC.

### 2.3 C Terminus Domain is Critical for sEV‐NID1's Oncogenic Ability

**FIGURE 3:**
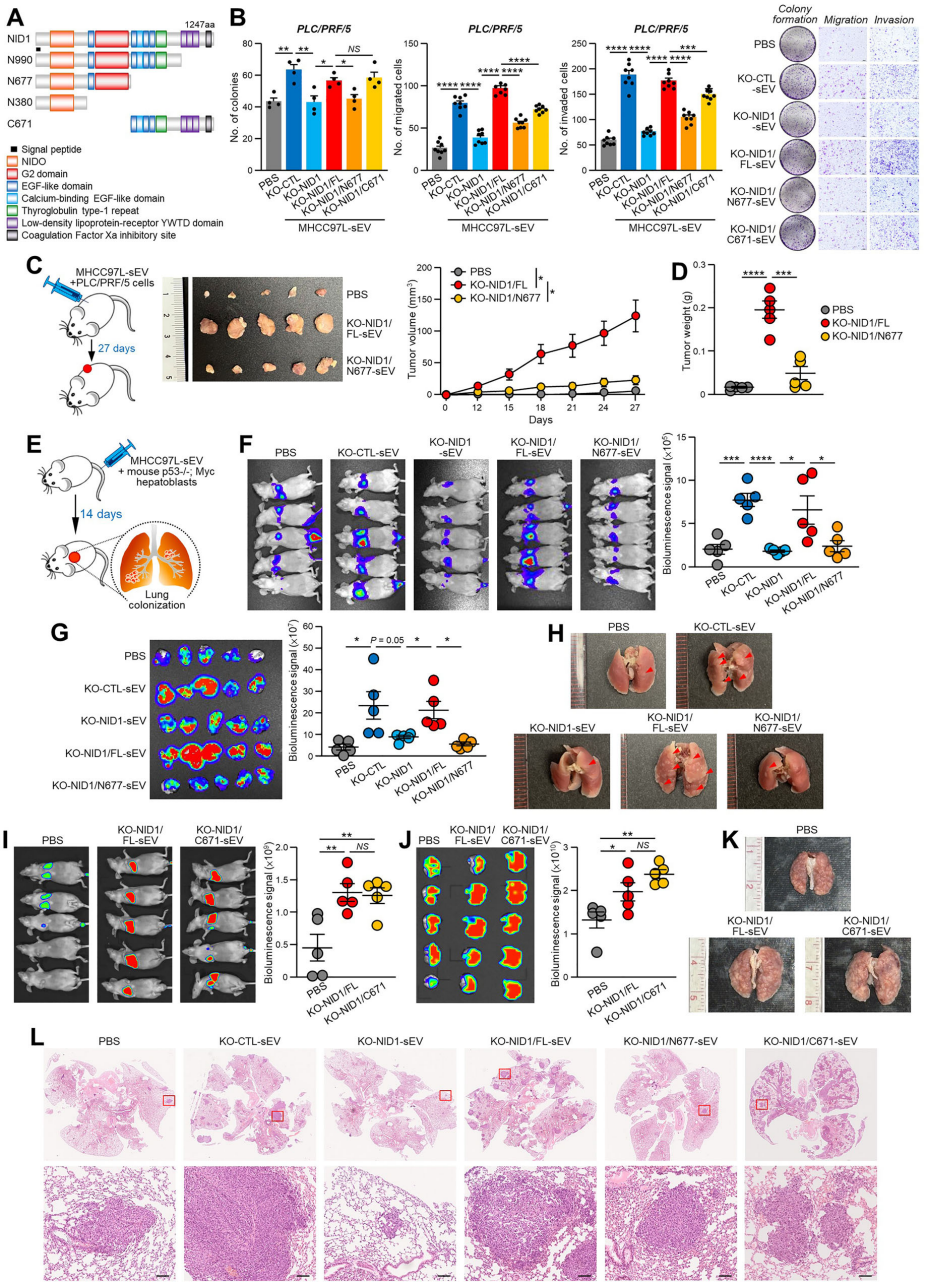
C terminus domain is critical for sEV‐NID1's oncogenic ability (A) A schematic diagram of the design of full‐length (FL), truncated mutants (N990, N677, N380) and C671 fragment of NID1. (B) PLC/PRF/5 cells were treated with PBS and sEV derived from MHCC97L KO‐CTL, KO‐NID1, KO‐NID1/FL, KO‐NID1/N677 and KO‐NID1/C671. Representative images and numbers of colonies (G) migrated (H) and invaded (I) cells were shown and quantified. (C–D) A scheme of a subcutaneous xenograft model(*left*), PLC/PRF/5 cells were co‐injected with PBS, FL‐sEV, or N677‐sEV derived from MHCC97L‐KO mutants. Representative photographs of explanted tumours (*middle*) and tumour growth curves (*right*) were shown and quantified. Endpoint tumour weights (D) were shown and quantified. (E) A schematic diagram of lung‐colonization assay, luciferase‐labelled murine p53‐/‐;Myc hepatoblasts were co‐injected with PBS, FL‐sEV, or N677‐sEV derived from MHCC97L‐KO mutants. (F and I) Whole‐body bioluminescence images at day 14 (*left*) and corresponding bioluminescence signals (*right*) were shown and quantified. (G and J) Bioluminescence imaging of excised lung tissues (*left*) and quantification of luciferase intensity (*right*). (H and K) Representative photographs of excised lung images with metastatic nodules were shown. (L) Representative H&E‐stained lung sections were shown. Scale bar, 100 µm.

The carcinogenic effect of sEV‐NID1 prompted us to investigate the underlying oncogenic mechanism by identifying which domain of NID1 is critical. To do so, we created sEV‐targeting XPack^TM^ NID1 full‐length (FL) and truncated mutants (N990, N677 and N380) according to NID1's three globular domains (Zhou et al. 2022) in HCC cells, HLE and Hep3B (Figure 3A and Figure S2A). Successful establishment of stable cells and sEV were validated for their purity, integrity and size (Figure S2A–C). The sEV derived from FL and mutant NID1 showed no obvious difference in these properties. Treatment of sEV derived from these cells showed that the loss of C terminus 671–1247 region significantly impeded sEV‐NID1's oncogenic ability, as indicated by significant reduction of colony formation, migration and invasion ability in N677‐sEV (Figure S3A–C). We further extended the examination on MHCC97L KO‐NID1 cells by overexpressing FL, N677 and C671 mutants of NID1 (Figure 3A and Figure S4). Notably, it was demonstrated that overexpression of NID1 FL and C terminus domain (C671) could rescue carcinogenic effect of sEV from KO‐NID1, as represented by enhanced colony formation, migration and invasion abilities, but sEV derived from N677 was incapable of doing so (Figure 3B, Figure S5). We then investigated whether loss of NID1 C‐terminus domain showed altered promotive effect of sEV *in vivo*. Using a subcutaneous xenograft model, we co‐injected PLC/PRF/5 cells with PBS, FL‐sEV or N677‐sEV derived from KO‐NID1 cells. We observed more aggressive tumour growth when cells were treated with FL‐sEV, but such enhancement was decreased when cells were treated with PBS or N677‐sEV (Figure 3C,D). We have previously reported that sEV‐NID1 facilitated extrahepatic metastasis (Mao et al. 2020). Therefore, we assessed whether N677‐sEV would display reduced effect in promoting metastasis. Luciferase‐labelled murine p53‐/‐;Myc hepatoblasts were co‐injected with various sEV in an experimental metastasis model (Figure 3E). Notably, treatment with KO‐CTL‐sEV and KO‐NID1/FL‐sEV showed enhanced lung colonization abilities (Figure 3F–H). However, in mouse groups treated with PBS and KO‐NID1‐sEV, such promotive effect was not observed. Overexpression of N677‐sEV was unable to rescue the oncogenic ability as well. Using the same mouse model, KO‐NID1/C671‐sEV was sufficient to promote metastasis as KO‐NID1/FL‐sEV (Figure 3I–K), suggesting the C terminus domain functions as the critical oncogenic region for sEV‐NID1. Immunohistochemical staining confirmed the presence of metastatic lesions in excised lung tissues (Figure 3L).

### 2.4 sEV‐NID1 C Terminus Domain Interacts With TGM3 in Recipient Cells

**FIGURE 4:**
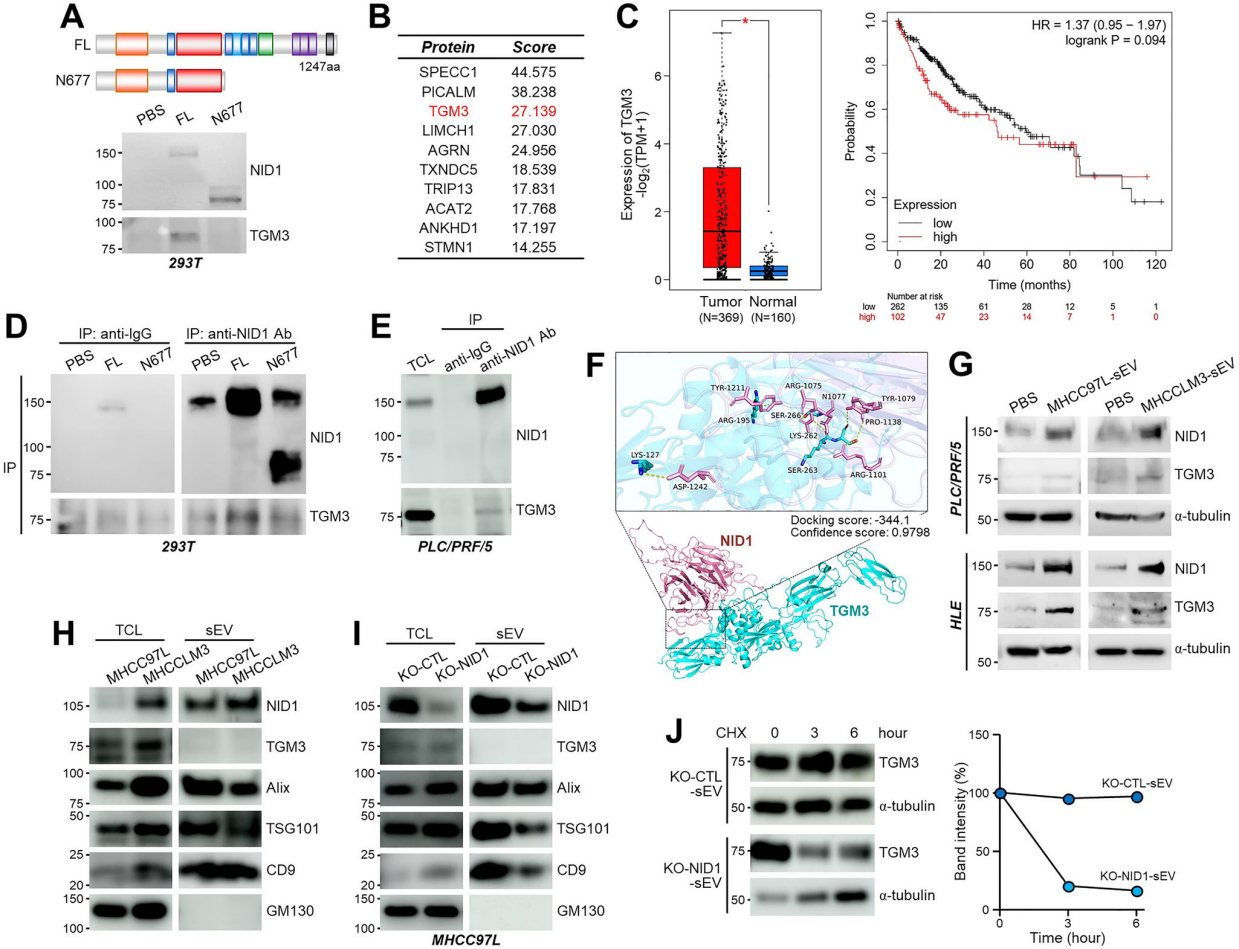
sEV‐NID1 C terminus domain interacts with TGM3 in recipient cells (A) A schematic diagram showing the structure of NID1‐FL and NID1‐N677. Immunoblotting of NID1 showing successful establishment of NID1‐FL and NID1‐N677 in 293T cells. (B) The top 10 most abundant proteins identified in 293T NID1‐FL pull‐down products, but not in CTL and NID1‐N677. The score is correlated with Q‐value. (C) The relative expression of TGM3 in in tumour (*n* = 369) than normal tissue samples (*n* = 160) from the TCGA‐LIHC cohort (*left*). Kaplan–Meier plot shows the overall survival between patients with high and low TGM3 expression in the TCGA‐LIHC cohort (*right*). (D) Cell lysates of 293T cells expressing FL and N677 were immunoprecipitated using anti‐IgG or anti‐NID1 antibody followed by immunoblotting for NID1 and TGM3 expressions. (E) Cell lysates of PLC/PRF/5 cells were immunoprecipitated with anti‐IgG or anti‐NID1 antibody followed by western blot analysis of NID1 and TGM3 expressions. (F) Binding analysis of NID1 and TGM3 was predicted using HDOCK. Interactions between NID1 and TGM3 interface residues were predicted by PyMOL. (G) PLC/PRF/5 and HLE cells were treated with sEV derived from MHCC97L and MHCCLM3 cells respectively. Cell lysates were subjected to immunoblotting. (H) Immunoblotting of TGM3 expression in total cell lysate (TCL) and sEV of MHCC97L and MHCCLM3 cells. (I) Immunoblotting of TGM3 expression in TCL and sEV of control (KO‐CTL) and NID1 knockout (KO‐NID1) cells established in MHCC97L cells. (J) Analysis of TGM3 expression in HLE cells treated with cycloheximide (CHX), KO‐CTL sEV and KO‐NID1‐sEV by western blotting.

Since NID1 mainly functions as a linker protein (Jeyagaran et al. 2025), it raises the possibility that its oncogenic effect may be regulated by interacting with other protein binding partners. Therefore, to understand NID1's interacting network, we established HEK293 CTL, NID1‐FL and NID1‐N677 stable clones and performed co‐immunoprecipitation (co‐IP) coupled to mass spectrometry analysis (Figure 4A). Compared to CTL and NID1‐N677, 25 proteins were found to be uniquely upregulated (unique peptides > 2, score > 11.1) in NID1‐FL (Figure 4B and Table S2). Among the differentially expressed co‐IP proteins, we selected one of the top candidates, TGM3, for further analysis since it been shown to promote HCC (Hu et al. 2020; Wu et al. 2022). Analysis of TCGA dataset revealed that a significant number of HCC patients exhibited higher levels of TGM3 in tumour (*n* = 369) than normal tissue samples (*n* = 160). TGM3 was also correlated with unfavourable overall survival in Kaplan–Meier analysis, highlighting the clinical significance of TGM3 in HCC (Figure 4C). We further validated TGM3's association with NID1 by immunoblotting, which showed an elevated TGM3 expression in NID1‐FL cell lysate as well as co‐IP samples in HEK293 cells (Figure 4D). The interaction between endogenous NID1 and TGM3 was further revealed in PLC/PRF/5 cells (Figure 4E). In silico analysis further predicted high‐affinity binding between NID1 and TGM3 (Docking score: −344.1; confidence score: 0.9798) (Figure 4F and Table S3). Notably, it was estimated that TGM3's N60‐N324 region would possibly bind to NID1's N818‐N1242 via several hydrogen bond interactions, which further implied reduction of TGM3 level upon loss of C‐terminus domain. PLC/PRF/5 and HLE cells treated with sEV exhibiting high NID1 expression derived from metastatic cells (MHCC97L‐sEV and MHCCLM3‐sEV) showed enhanced NID1 and TGM3 protein levels (Figure 4G). It is shown that TGM3 was not expressed in sEV (Figure 4H), indicating that the observed elevation in TGM3 is due to the stabilization of endogenous TGM3 in recipient cells rather than direct transfer via sEV. Given the interaction between TGM3 and NID1, we hypothesized that the uptake of sEV‐NID1 may stabilize and enhance TGM3 protein expression in recipient cells. To test this, we assessed TGM3 stability in cells treated with sEVs from KO‐CTL and KO‐NID1 cells in the presence of cycloheximide (Figures 4I and 4J). Cells treated with KO‐CTL‐sEV retained higher TGM3 protein levels compared to cells treated with KO‐NID1‐sEV, indicating that sEV‐NID1 stabilizes TGM3 protein against degradation. Finally, qPCR analysis showed no significant difference in TGM3 transcript levels between cells treated with KO‐CTL‐sEV and KO‐NID1‐sEV (Figure S6), suggesting that sEV‐NID1 influences TGM3 at the translational expression, rather than transcriptional level.

### 2.5 TGM3 is the Downstream Activator of sEV‐NID1 That Promotes HCC Aggressiveness

**FIGURE 5:**
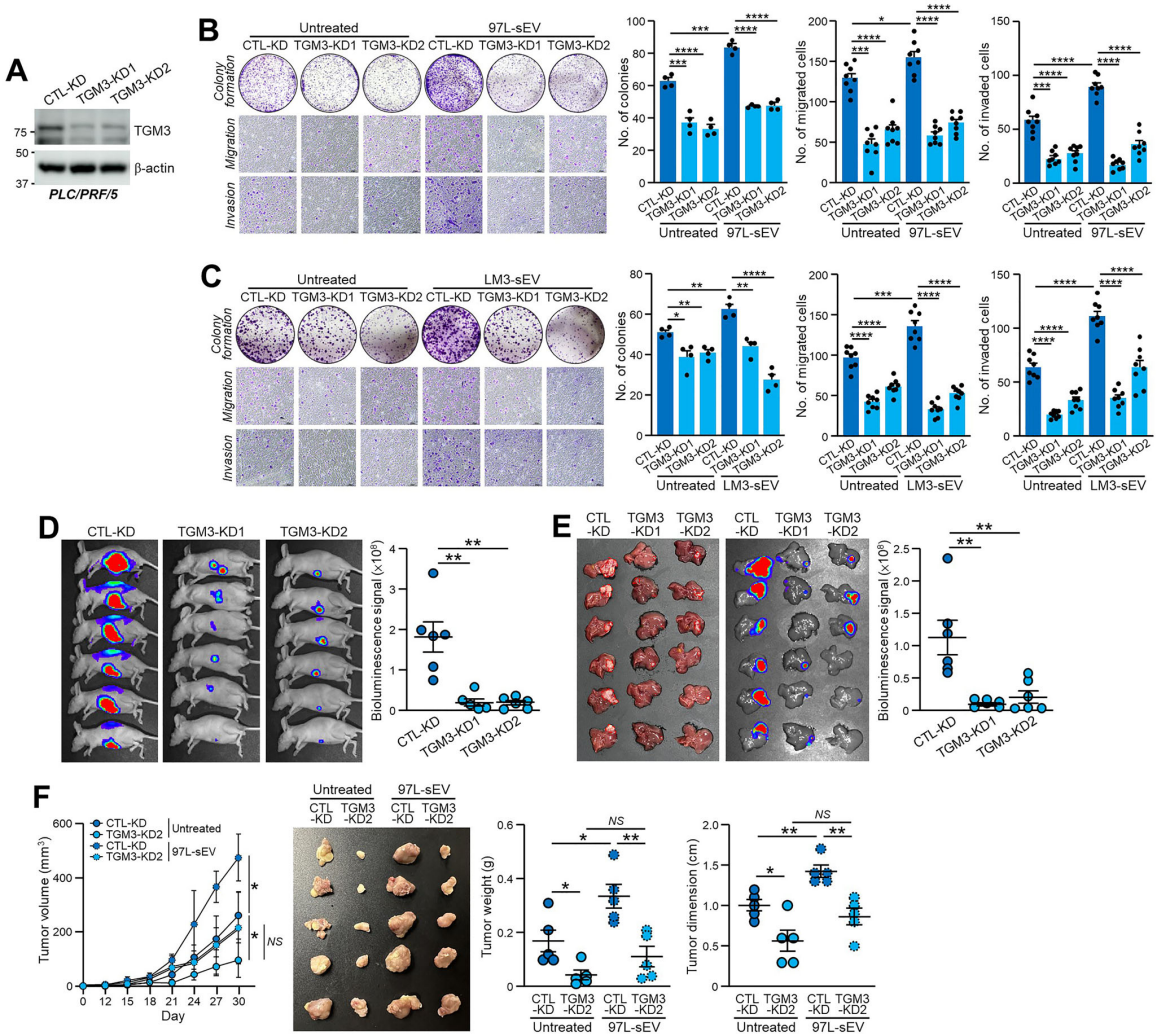
TGM3 is the downstream activator of sEV‐NID1 that promotes HCC aggressiveness (A) Immunoblotting of TGM3 and ⍺‐tubulin in CTL‐KD and TGM3‐KD cells established in PLC/PRF/5. (B–C) PLC/PRF/5 CTL‐KD, TGM‐KD‐1 and TGM‐KD‐2 were treated with PBS, 97L‐sEV(B) and LM3‐sEV (C) respectively. Treated cells were then subjected to colony formation(upper), migration(middle) and invasion(bottom) assays. Representative diagrams were shown and number of cells were quantified. (D–E) PLC/PRF/5 CTL‐KD and TGM3‐KD cells (e6) were orthotopically implanted to nude mice liver. After 6 weeks post‐implantation, bioluminescence signals of whole mice (D) and excised livers tissues(E) were imaged and quantified. (F) PLC/PRF/5 CTL‐KD and TGM3‐KD cells (1.5e6), which were treated with PBS or 97L‐sEV, were subcutaneously injected into nude mice. After 30 days post‐injection, tumours were excised and imaged. Tumour weight and tumour dimension were measured.

Given the correlation between NID1 and TGM3, we sought to explore TGM3's functional role in HCC carcinogenesis. We established stable TGM3 knockdown clones (TGM3‐KD1 and TGM3‐KD2) in 2 recipient HCC cells (PLC/PRF/5 and HLE) (Figure 5A and Figure S7A). Reduction in cellular TGM3 level significantly hampered the oncogenic properties of HCC cells, as indicated by reduced colony formation, migration and invasion abilities. Moreover, it also reduced the responsiveness of recipient cells to the promotive effect of 97L‐sEV (Figures 5B,C and S7B,C). To further demonstrate the *in vivo* role of TGM3 in carcinogenesis, we performed orthotopic liver implantation, in which PLC/PRF/5 TGM3‐KD cells exhibited a remarkedly slower tumour growth rate than CTL‐KD cells (Figure 5D, E). Upon 97L‐sEV treatment, CTL‐KD cells showed more aggressive cancer behaviour, but such promotive effect was significantly inhibited in TGM3‐KD cells (Figure 5F). In agreement with the *in vitro* model, subcutaneous injection of TGM3‐KD cells also resulted in smaller tumour development and reduced responsiveness to 97L‐sEV treatment as compared to CTL‐KD cells. Furthermore, cells treated with and without KO‐NID1‐sEV yielded comparable colonies, migrated and invaded cells, demonstrating that NID1 is the functional component of 97L‐sEV. In addition, the pro‐oncogenic function of sEV‐NID1 was fully abrogated in TGM3‐KD cells (Figure S8). Collectively, these results suggest that TGM3 functions as the downstream effector of sEV‐NID1 and loss of TGM3 expression alleviates sEV‐NID1's oncogenic effects.

### 2.6 Loss of TGM3 Leads to Integrin Dysregulation and Hippo Pathway Activation

**FIGURE 6:**
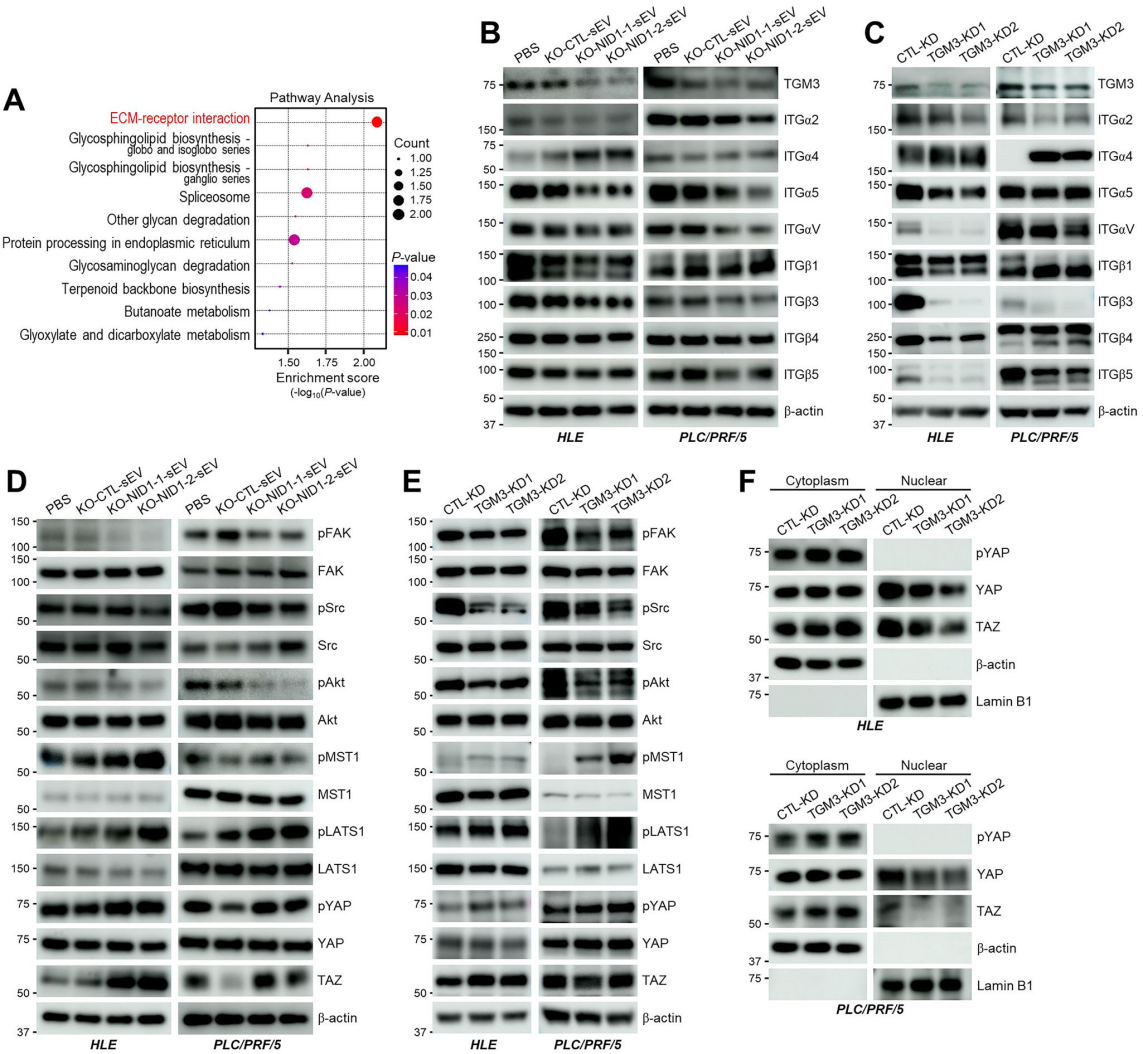
Loss of TGM3 leads to integrin dysregulation and Hippo pathway activation (A) Kyoto Encyclopedia of Genes and Genomes (KEGG) analysis of NID1‐FL upregulated binding proteins (unique peptides > 2; score > 11.1) showed enhanced ECM‐receptor interaction (red). (B) Western blot analysis of TGM3 and integrins (⍺2, ⍺4, ⍺5, ⍺V, β1, β3, β4 and β5) in HLE and PLC/PRF/5 cells treated with or without sEV derived from MHCC97L KO‐CTL, KO‐NID1 cells. (C) Immunoblotting of TGM3 and integrins in HLE and PLC/PRF/5 CTL‐KD and TGM3‐KD cells. (D) Immunoblotting of the phosphorylation of FAK‐Src‐Akt signalling pathway, Hippo kinase cascade (MST1 and LATS1) and transcriptional coactivator (YAP and TAZ) in HLE and PLC/PRF/5 cells treated with or without sEV derived from MHCC97L KO‐CTL, KO‐NID1 cells. (E) Western blot analysis of FAK‐Src‐Akt signalling pathway, Hippo kinase cascade as well as YAP and TAZ translational coactivator in HLE and PLC/PRF/5 CTL‐KD and TGM3‐KD cells. (F) Cellular fractionation of PLC/PRF/5 CTL‐KD and TGM3‐KD cells and analysis of YAP and TAZ.

Similar to NID1, TGMs are known to cross‐link with various ECM proteins (Martinez et al. 1994). In fact, analysis of the NID1‐FL upregulated binding proteins (unique peptides > 2; score > 11.1) using the Kyoto Encyclopedia of Genes and Genomes (KEGG) revealed ECM‐receptor interaction to be strongly enriched (Figure 6A). Given the fact that the integrin family plays an important role in ECM orientation, we examined the expression of various integrins in HLE and PLC/PRF/5 cells affected by NID1 delivered by sEV. We noticed dysregulation of various integrins, including reduction in ⍺2, α5, ⍺V, mature β1, β3, β4 and β5 and an enhancement in ⍺4 in both cell lines treated with KO‐NID1‐sEV compared to cells treated with KO‐CTL‐sEV (Figure 6B). Similar dysregulation of integrins was noted in cells upon the loss of TGM3 (Figure 6C). Integrins have been shown to mediate various oncogenic pathways, including YAP/TAZ activities (Warren et al. 2018). From our immunoblotting result, the loss of ⍺2β1 integrin expression prompted us to investigate whether the subsequent YAP oncogenic signalling pathway was inhibited. Our immunoblotting results revealed that compared to cells treated with KO‐CTL‐sEV, KO‐NID1‐sEV treatment led to reduced phosphorylation of Src (Tyr416), FAK (Tyr397) and Akt (Ser473). Subsequently, this caused the activation of MST1 and LATS1, which repressed YAP transcriptional activity by phosphorylation at Ser127, leading to its sequestration in the cytoplasm (Figure 6D). Additionally, we observed that TGM3 knockdown in cells reduced the activity of the FAK/Src/Akt pathway and promoted the activation of MST1/LATS1 (Figure 6E). Cellular fractionation demonstrated that the inactive, phosphorylated form of YAP was exclusively detected in the cytoplasmic fraction. Additionally, TGM3 knockdown cells exhibited reduced nuclear levels of both YAP and TAZ (Figure 6F). Together, these findings demonstrate that TGM3 level is essential for sEV‐NID1‐induced integrin activation of YAP/TAZ pathway, leading to their sequestration in the cytoplasm.

### 2.7 Therapeutic Potential of Targeting NID1 With Monoclonal Neutralizing Antibody

**FIGURE 7:**
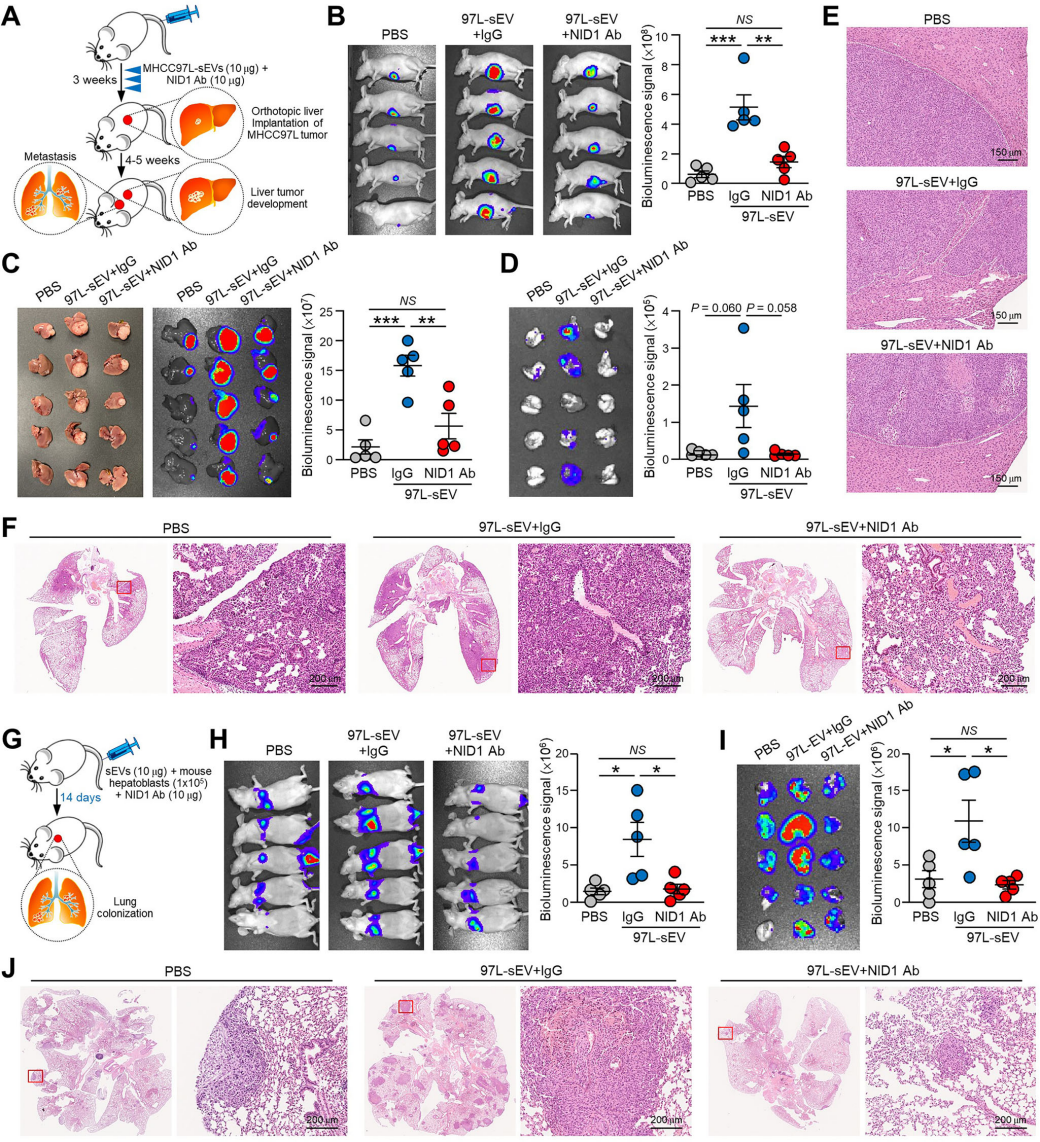
Therapeutic potential of targeting NID1 with monoclonal neutralizing antibody (A) An illustration of sEV education model in combination with anti‐NID1 mAb treatment. Prior to orthotopic liver implantation of MHCC97L cells (e^6^), PBS, MHCC97L sEV and anti‐NID1 mAb (10 µg) were administrated intravenously for 3 consecutive weeks. (B–D) After 6 weeks post‐implantation, bioluminescence signals of whole mouse (B), excised tumour tissues (C) and lung tissues (D) were captured and quantified. (E) Representative H&E‐stained liver tumour and surrounding tissue were shown. Scale bar, 150 µm. (F) Representative H&E‐stained lungs and tumour nodules were shown. Scale bar, 200 µm. (G) An illustration of lung colonization model in combination with anti‐NID1 mAb treatment. Mice were injected with murine p53‐/‐;Myc hepatoblasts via tail vein, together with the treatment of PBS, MHCC97L sEV and anti‐NID1 mAb (10 µg) respectively. (H–I) Two weeks post‐injection, bioluminescence signals of whole mouse (H) and excised lungs(I) were imaged and quantified. (J) Representative H&E‐stained lungs and tumour nodules were shown. Scale bar, 200 µm.

**FIGURE 8:**
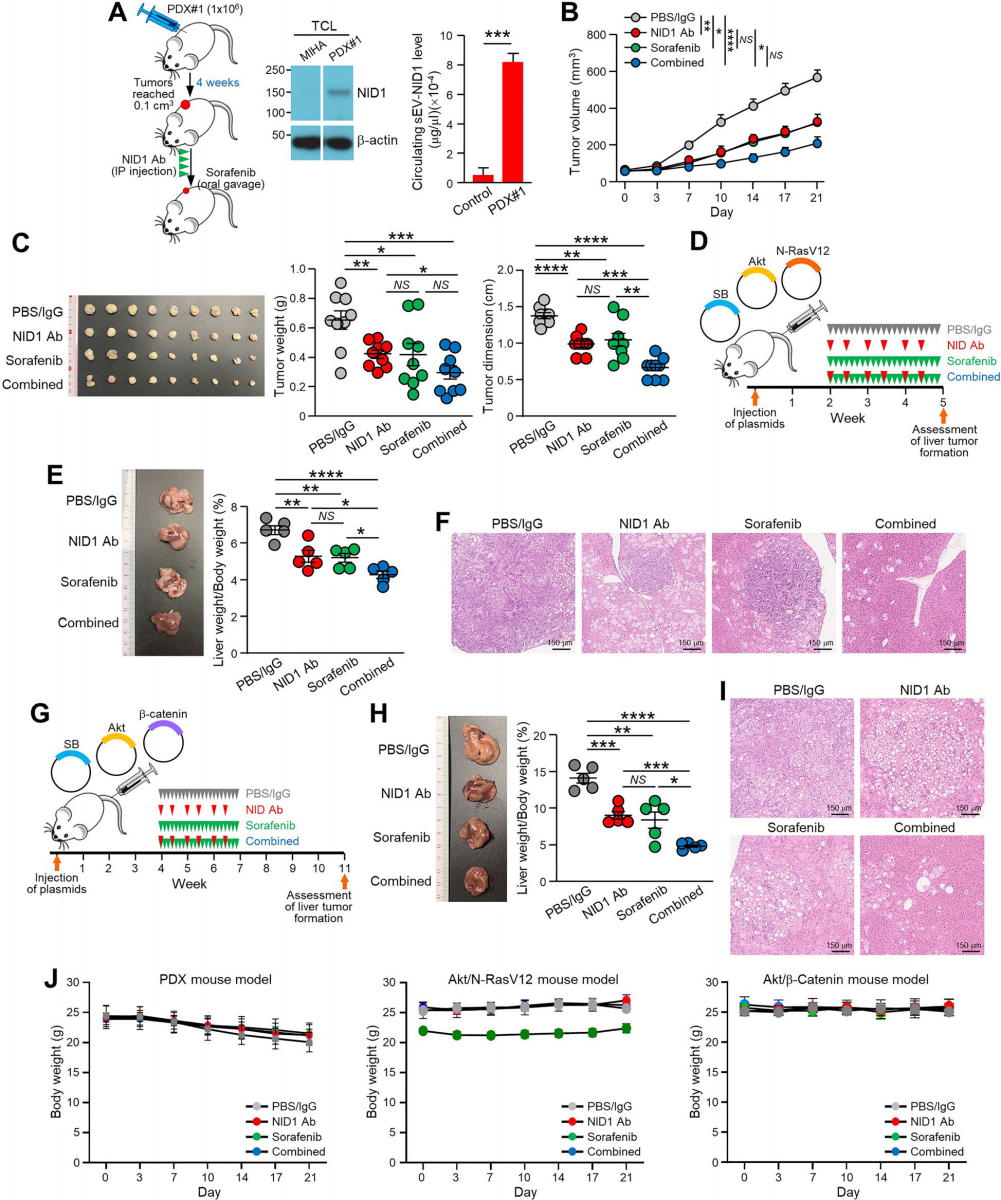
Combination Anti‐NID1 mAb and Sorafenib therapy potently suppresses HCC (A) An illustration of the subcutaneous injection of patient‐derived xenograft (PDX#1) (e6) to nude mice. After the tumour has reach 0.1 cm^3^, mice will be treated with DMSO/IgG, anti‐NID1 mAb, sorafenib and combination treatment respectively. Immunoblotting of NID1 and β‐actin of total cell lysate from normal hepatocyte MIHA and PDX#1. Circulating sEV‐NID1 level was measured from serum isolated from nude mice injected with/without subcutaneous PDX#1 tumour. (B) Tumour volume was monitored and measured twice per week. (C) At the endpoint, tumours were excised and shown. Tumour weight and dimension were measured. (D) An illustration of the delivery of oncogenic plasmids, including sleeping beauty transposon (SB), Akt and NRasV12, via hydrodynamic tail vein injection. (E) Representative images of the excised liver tissues were shown. Liver to body weight (%) was measured and calculated. (F) Representative H&E‐stained liver tumours and surrounding tissues were shown. Scale bar, 150 µm. (G) An illustration of the delivery of oncogenic plasmids, including sleeping beauty transposon (SB), Akt and β‐catenin, via hydrodynamic tail vein injection.(H) Representative images of the excised liver tissues were shown. Liver to body weight (%) was measured and calculated. (I) Representative H&E‐stained liver tumours and surrounding tissues were shown. Scale bar, 150 µm. (J) Body weight of the up mentioned 3 mouse models were monitored and measured twice per week.

Based on our previous findings, we have shown that monoclonal anti‐NID1 antibody (anti‐NID1 mAb) can target NID1 in multiple cancer types (Xue et al. 2025). We therefore hypothesized that anti‐NID1 mAb could work against sEV‐NID1 mediated oncogenesis as a potential therapeutic option against HCC. We first evaluated the inhibitory effect of anti‐NID1 mAb against sEV‐NID1 *in vitro*. We observed anti‐NID1 mAb could inhibit the promotive effect of sEV‐NID1 derived from metastatic HCC cells and late‐stage HCC patient's serum (L‐HCC‐sEV) (Figure S9). We further extended our evaluation of anti‐NID1 mAb's therapeutic functions in various HCC‐relevant animal models. We first employed a sEV education model by repeated inject sEV‐NID1, with or without anti‐NID1 mAb co‐treatment, intravenously before orthotopically implanted HCC tumour seed to the liver (Costa‐Silva et al. 2015) (Figure 7A). Co‐treatment of anti‐NID1 mAb against sEV‐NID1 impeded the oncogenic effects of 97L‐sEV, as demonstrated by reduced tumour growth and incidence of lung metastasis (Figure 7B–F). The lung colonization model also has a similar trend, in which hepatoblasts co‐injected with 97L‐sEV and L‐HCC‐sEV had markedly greater number of metastatic lesions. However, addition of anti‐NID1 mAb can inhibit the promotive effects of these cancer‐derived sEV (Figure 7G–J, Figure S10A–E). Since sEV treatment would lead to upregulation of cellular level (Figure 1D), we continued to examine the therapeutic effect of anti‐NID1 mAb against cellular NID1, using HCC mouse models with high NID1 tumour expression. A patient‐derived xenograft (PDX), which has elevated NID1 cellular expression and circulating sEV‐NID1 level in implanted mice, was therefore employed (Figure 8A). Mice were treated with anti‐NID1 mAb in combination with sorafenib, a current first line chemotherapy for HCC patients. We observed that the combination of anti‐NID1 mAb and sorafenib brought the most significant tumour inhibitory effect, as compared to DMSO/IgG or single treatment of sorafenib and anti‐NID1 mAb (Figure 8B, C). Similar therapeutic efficacy of anti‐NID1 mAb was observed in 2 HDTVi‐induced tumour models, Myr‐AKT/N‐RasV12 (Figure 8D–F) and Myr‐AKT/N90‐β‐catenin (Figure 8G–I). These treatments were well tolerated with no obvious difference in body weight among mouse groups (Figure 8J). Based on our observation, we propose a potential therapeutic strategy by blocking both sEV‐NID1 and cellular NID1 using anti‐NID1 mAb.

## 3 Discussion

NID1 is a sulphated glycoprotein, which is important for the BM organization. Multiple proteins, including laminin‐1, type IV collagen, and collagen type IV, were identified as NID1's interacting partners (Pujuguet et al. 2000). NID1 overexpression was found to be associated with tumour aggressiveness and poor prognosis in multiple cancers (Mao et al. 2020; Urooj et al. 2020; Zhou et al. 2017). sEV are lipid‐membrane‐enclosed vesicles with sizes ranging from 30–150 nm (Jeppesen et al. 2019). They are vital in cellular communication and molecular transfer (Sancho‐Albero et al. 2019). Hence, elucidating the molecular mechanisms that contribute to sEV‐NID1 oncogenic ability will be crucial. Our previous studies have unveiled the contribution of sEV‐NID1 in promoting extrahepatic metastasis (Mao et al. 2020). Here, we demonstrated that treatment of sEV‐NID1 led to upregulated NID1 cellular expression in recipient cells and is associated with more aggressive oncogenic features. Conversely, from knockout experiments, it was shown that reduction in cellular‐ and sEV‐NID1 lost such promotive effect both *in vitro* and *in vivo*, which is consistent with our previous study using shRNA. Thus, this further validated the contribution of NID1 in HCC progression. However, the mechanism of NID1's upregulation in sEV and its downstream activator remains unclear. Having a better understanding of the contributing factors for sEV‐NID1's overexpression and its oncogenic mechanism in HCC will be crucial for designing better anti‐NID1 targeted therapies.

Matrix stiffening is a process caused by ECM remodelling, regulated by factors such as enzymatic remodelling, fibroblast activation, and cross‐linking of ECM proteins (Mai et al. 2024). It has been reported that an increment in matrix stiffness would regulate sEV's (exosomes) secretion and protein level (Wu et al. 2023). In breast cancer, stiff ECMs tuned sEV‐enriched thrombospondin‐1 to induce pro‐invasion properties (Patwardhan et al. 2021). Similar behaviour was also observed in pancreatic ductal adenocarcinoma. A stiffer matrix was shown to induce cancer‐associated fibroblasts to secrete sEV hyperactively, which contributed to enhanced proliferation and chemoresistance (Xiao et al. 2022). Matrix stiffening is common in the liver due to processes such as fibrosis and cirrhosis (Wells 2005). In particular, hepatic stellate cells were shown to transdifferentiate and deposit excessive matrix and were associated with liver fibrosis (Kostallari et al. 2022). Our study showed that HCC cells cultured on a stiffer matrix released a significantly higher concentration of sEV and enhanced expression of sEV positive markers. Our *in vitro* and *in vivo* functional assays revealed stiff‐sEV had a higher tumorigenic ability as well. In addition, we also found that NID1 level was positively correlated with ECM stiffness, as shown by enhanced NID1 mRNA transcripts in cells cultured on 8 kPa matrix as compared to 0.5 kPa. Interestingly, our immunoblotting results revealed that NID1 was selectively upregulated in sEV, but not in cellular form, suggesting that stiff matrix‐induced NID1 was predominantly secreted extracellularly. However, further studies are required to delineate the underlying mechanism of this selective induction.

Being an important linker, NID1 has been reported to interact with multiple ECM constituent proteins, allowing the formation of a mobile yet stabilized macromolecular structure (Kadoya et al. 2022). Despite extensive studies on NID1's binding partners, which domain is critical for sEV‐NID1's oncogenesis remains unclear. In order to search for the critical sEV‐NID1 domain, we established sEV‐targeting NID1 FL and mutant stable clones, which were designed based on NID1's three globular domains (G1‐G3). Our data found that the loss of C‐terminus region (N677‐N1247) most significantly hampered sEV‐NID1's oncogenic ability both *in vitro* and *in vivo*. We further showed that the restoration of the C‐terminus region (C671) partially rescued sEV‐NID1's tumour promotive effect. The C‐terminus domain is comprised of a rod‐like domain and G3 domain. Multiple proteins have been identified to interact with the C‐terminus region, including laminin γ1 chain and fibulin‐2 (Takagi et al. 2003). Using co‐IP MS to analyse NID1‐FL and N677 mutants’ pull‐down products, we identified multiple proteins involved in cell adhesion and structural development, namely PICALM, LIMCH1, TRIP13, to be dysregulated upon the loss of the C‐terminus domain. The well‐studied G3 binding partner, laminin subunit alpha‐5, was also significantly reduced. Among all the pull‐down candidates, we identified TGM3 to be one of the top NID1 C‐terminus domain binding partners. TGM3 belongs to the transglutaminase family, which are cross‐linking enzymes between free amine groups and γ‐carboxamide groups in a Ca^2+^‐dependent manner (Eckert et al. 2014; Nurminskaya and Belkin 2012). Our TCGA and survival analysis revealed that TGM3 mRNA level was upregulated in HCC and was correlated with poor survival. Consistently, recent studies have also shown the involvement of TGM3 in HCC, which revealed overexpression of TGM3 was associated with epithelial‐mesenchymal transition via the activation of AKT/GSK3β/β‐catenin pathway, leading to poor prognosis after curative resection (Hu et al. 2020). Using multiple database analysis, TGM3 was also found to be correlated with tumour stages, mutational burden, microsatellite instability and decreased immune cell infiltration in HCC (Zhang et al. 2023). In addition, our co‐IP and immunoblotting result then validated the upregulation of TGM3 in both FL cells and pull‐down products, while N677 cells did not observe such elevation. We also showed that treatment of sEV‐NID1 led to higher NID1 as well as TGM level in recipient cells. Moreover, our in‐silico analysis further predicted high binding affinity between TGM3 with NID1's C‐terminus domain These results raise the possibility that TGM3 may function as the downstream activator of sEV‐NID1 via binding with the C‐terminus domain.

To examine whether TGM3 act as the downstream regulator, we employed two independent shRNAs and demonstrated that loss of TGM3 inhibited HCC oncogenesis. We further showed that TGM3 is required for sEV‐NID1's functionality, as shown by hampered sEV‐NID1 oncogenic abilities when treated to TGM3‐KD cells. Pathway analysis of NID1's C‐terminus domain binding partners revealed ECM‐receptor interaction to be profoundly enriched. As a matter of fact, it has been reported that TGMs played non‐enzymatic role. For instance, TGMs presented on the cell surface were shown to adhere with various ECM members, ranging from fibronectin (FN) to integrins (Akimov et al. 2000; Belkin et al. 2005; Zemskov et al. 2006). Through these ligand bindings, TGMs were able to mediate different oncogenic pathways. In ovarian cancer, TGMs were found to form complex with integrin‐linked kinase in the presence of FN matrix, leading to the activation of the Wnt/β‐catenin pathway (Condello et al. 2022). Similar situation was also observed in squamous cell carcinoma, when TGMs mediated cancer stem cell survival via interaction with ⍺6/β4 integrin (Fisher et al. 2016). Hence, we tested whether loss of TGM3 would affect ECM proteins expression. Accordingly, our data showed reduced FN and various integrins levels in TGM3‐KD cells, demonstrating TGM3's interactions with other ECM components. Next, we identified the Hippo pathway as a main mechanism regulated by TGM3‐⍺2β1 integrin interaction. The Hippo signalling is an important regulator of cell proliferation, migration and survival (Kim et al. 2018). When the Hippo kinase cascade (Mst1/2) is activated, phosphorylation of the YAP/TAZ transcription coactivators will lead to cytoplasmic retention and degradation, thus inhibiting downstream transcriptional activities (Sohn et al. 2016). In the liver, the Hippo pathway has been shown to be important regulator of liver growth and tumorigenesis, of which overexpression and nucleus translocation of YAP result in hepatomegaly and tumour formation (Liu et al. 2020). Our findings demonstrated that reduction in TGM3 led to FAK‐Src‐AKT inactivation, which is a major integrin regulated signalling pathway (Mitra and Schlaepfer 2006). In addition, we also observed activation of Hippo kinases MST1 and subsequent phosphorylation of LATS1. This resulted in phosphorylation of the YAP and inhibition of downstream transcriptional activities. Altogether, these data strengthen the role TGM3 as NID1's effector in HCC oncogenesis via regulating integrin mediated Hippo pathway regulation.

Finally, we evaluated whether blockade of sEV‐NID1 has therapeutic outcomes. We have previously developed a homemade anti‐NID1 mAb using hybridoma technology, which showed anti‐tumorigenic effects against four types of cancers (Xue et al. 2025). We showed that targeting sEV‐NID1 using mAb could effectively inhibited colony formation, migration and invasion capacity. Immunoblotting analysis further showed that anti‐NID1 mAb reduced both NID1 and TGM3 upregulation upon sEV‐NID1 treatment. Similar results were also observed in various animal models, which exhibited delayed tumour formation and extrahepatic lung metastasis when anti‐NID1 mAb was administered. These results support the blockade of NID1 using mAb as a potential preclinical treatment strategy. How the antibody function against sEV‐NID1, the antibody's targeted mechanism and tissue distribution still warrant further investigation.

In conclusion, our data reinforce the role of sEV‐NID1 in hepatocarcinogenesis and identify the C‐terminus as the critical domain, of which matrix stiffness is one of the contributing factors for sEV‐NID1 overexpression. We further identify TGM3 as the downstream effector of sEV‐NID1 and is responsible for mediating integrin‐Hippo signalling regulation. The proof of concept of targeting sEV‐NID1 using a homemade anti‐NID1 antibody was demonstrated in various HCC‐specific animal models, suggesting its potential as a therapeutic strategy for HCC tumours. However, in this study, it remains an open question why matrix stiffness leads to enhanced sEV‐NID1 expression; whether this is caused by enhanced packaging of NID1 into sEV remains to be explored. Another limitation of this study is the lack of an appropriate loading marker for sEV quantification. Despite the fact that we have tried to quantify sEV protein by Coomassie blue staining, this assay is not specific to sEV alone. Moreover, in this study, we showed that sEV‐NID1 interacts with TGM3 by co‐IP and in silico analysis. More in‐depth structural studies may require by the use of cryogenic electron microscopy. Finally, the administration of a homemade anti‐NID1 antibody hampered HCC oncogenesis in various animal models. Yet, we have not studied the mechanistic effect of this antibody, and whether it will inhibit the NID1‐TGM3‐mediated Hippo pathway activation.

## 4 Methods and Materials

### 4.1 Cell Culture

Human normal immortalized hepatocyte MIHA was obtained from Jayanta Roy‐Chowdhury, Albert Einstein College of Medicine, New York. Human HCC cell lines PLC/PRF/5, human embryonic kidney (HEK) 293FT and 293T were purchased from the American Type Culture Collection (ATCC). Other non‐metastatic HCC cell lines, including HLE and Huh7, were purchased from the Japanese Collection of Research Bioresources (JRCB, Japan). Metastatic MHCC97L were provided by the Cancer Institute, Fudan University, China. Murine p53‐/‐; Myc hepatoblasts were kindly gifted by the Scott Lowe Lab at the Memorial Sloan Kettering Cancer Centre, New York. Hybridoma cells for the purification of anti‐NID1 mAb were provided by DiagnoVEX Therapeutics Limited. All cell lines were cultured according to the instructions by providers and were routinely screened for mycoplasma contamination.

### 4.2 Isolation of sEV

#### 4.2.1 From Cell Conditioned Medium

In order to isolate sEV from cell cultured conditioned medium, cells were seeded on petri dish at a confluency of 70%–80% and were allowed to culture overnight. After cells were attached, they were washed twice with PBS. Full culture medium supplemented with 10% sEV‐depleted FBS were then used to replenish the cells. After 72 h of culturing, cells would reach maximum confluency, and their conditioned medium was collected. The conditioned medium was then centrifuged at 3000 × g for 15 min at 4°C to remove dead cells. The supernatant was then collected and subjected to differential ultracentrifugation using SW45Ti Fixed‐Angle rotor (Beckman Coulter). In brief, 70 mL polycarbonate bottle with cap assembly were first topped up and balanced, followed by centrifugation at 20,000 × g for 30 min at 4°C. The supernatant was then filtered through 0.22 µm Steritop Vacuum Bottle Top Filter (Millipore) to remove microvesicles. Filtered medium was then centrifuged at 100,000 × g for 70 min at 4°C. Supernatant was discarded and the sEV pellet was washed with PBS and centrifuged at 100,000 × g for 70 min at 4°C. The sEV pellet was then resuspended in PBS for further usage.

#### 4.2.2 From Serum Samples

Whole blood was collected and placed at room temperature for 30 min, which allow blood clot to form. Afterwards, the blood sample was centrifuged at 1500 × g for 10 min at 4°C. Serum (supernatant) was then extracted from the clot. Serum samples (1 mL) were then topped up by PBS and subjected to the differential ultracentrifugation steps using SW45Ti Fixed‐Angle rotor (Beckman Coulter) as the aforementioned procedures of cell conditioned medium. Serum samples were collected at Queen Mary Hospital, Hong Kong. Ethical approval for the procedure (IRB reference number: UW 23‐264) was obtained from the Institutional Review Board of The University of Hong Kong/Hospital Authority Hong Kong West Cluster (HKU/HA HKW IRB). All experiments involving human samples were conducted in compliance with applicable ethical guidelines and regulations.

### 4.3 Animal Husbandry and Ethical Issues

BALB/cAnN‐nu (Nude), C57BL/6J and NID1‐KO mice were housed in the Centre for Comparative Medicine Research (CCMR) at the University of Hong Kong. The experimental procedures approved by CULATR 5579–20, 5376–20, 5217–19, 22–067 were conducted according to the guidelines approved by the Committee on the Use of Live Animals in Teaching and Research and Animal (Control of Experiments) Ordinance.

### 4.4 Development of NID1 Knockout (NID1‐KO) Mouse Model

NID1‐KO mouse model was created by CRISPR/Cas‐mediated genome engineering (Cyagen) on C57BL/6N strain. In brief, NID1's exons were identified, and 2–5 exons were selected as targeted sites. Cas9 and designed gRNA were then co‐injected into fertilized eggs. Homozygous KO mice were then identified by genotyping.

### 4.5 DEN/CCl_4_

On the day of administration, 1 mg N‐Nitrosodiethylamine (DEN)(Sigma‐Aldrich) solution was freshly added to 15 mL PBS. The mixture was injected intraperitoneally into 14‐day old male C57BL/6N WT and NID1‐KO mice at a dose volume of 15 µL/g. To prepare CCl_4_, 1 mL of CCl_4_ was dissolved in 9 mL olive oil (Sigma‐Aldrich). When the mice reached 8 weeks of age, CCl_4_/olive oil were injected intraperitoneally at a concentration of 0.2 mL/kg twice a week for 14 weeks consecutively. At the end point, mice were sacrificed, and liver tumours were collected for histological analysis.

### 4.6 Hydrodynamic Tail Vein Injection (HDTVi) Mouse Model

Plasmids (pT3‐EF1‐NRAS, pT3‐EF1⍺H‐myr‐Akt, pT3‐EF1⍺H N90‐beta‐catnin and PT2/C‐Luc/PGK‐SB13) were purchased from Addgene. Plasmid solution for injection was freshly prepared by adding 20 µg oncogenic plasmids and 1.6 µg Sleeping Beauty transposon into 3 mL filtered PBS. The mouse tail of 6–8 weeks old C57BL/6J mouse was then warmed for 10–20 s until the plasmid solution was injected via tail vein within 5–8 s. Bioluminescence signal from the liver tumours was measured and quantified by IVIS Spectrum *in vivo* imaging system (Perkin Elmer). At the end point, mice were sacrificed, and liver tumours were collected for histological analysis.

### 4.7 Lung Colonization Study

Murine p53‐/‐;Myc hepatoblasts (100,000) were resuspended in 100 µL PBS. The mixture was then injected intravenously via 6–8 weeks old nude mice tail vein. Bioluminescence signal of the lungs of the mice was visualized and quantified by IVIS Spectrum *in vivo* imaging system (Perkin Elmer). At the end point, mice were sacrificed, and lungs were collected for histological analysis.

### 4.8 Subcutaneous Tumour Xenograft

PLC/PRF/5 cells (3 × 1,000,000) were suspended in 100 µL PBS and Matrigel Matrix mixture (1:1), which was then injected subcutaneously into the flank of 4–6 weeks old male nude mice. After 14 days after injection, tumour size was measured using a caliper twice every week. Body weight was also monitored. At the end point mice were sacrificed and tumours were excised and weighted. Tumour volume was calculated as ½ × length × width^2^.

### 4.9 Patient Tumour‐Derived Xenograft (PDX)

The PDX tumour employed in this study was kindly gifted by Irene Ng, The University of Hong Kong. To expand the PDX xenograft, the PDX tumour was first cut and minced into small pieces. Cold DMEM/F12 medium (10 mL) supplemented with 100 µL DNase I (Sigma‐Aldrich) and 25 µL Liberase (Roche) was added to the tumour mince. The mixture was then allowed to shake gently for 30 min at 37°C. Afterwards, the dissociated tumour mixture was filtered through a 100 µm strainer. The flow through was topped up to 50 mL with cold DMEM/F12 medium and centrifuged at 700 rpm for 5 min at 4°C. The supernatant was discarded, and the mixture was washed again with 50 mL cold DMEM/F12 medium until no blood cells/debris remained. Cells were resuspended in DMEM/HG and counted with hemacytometer. PDX dissociated cells (e^6^) were then mixed with 100 µL PBS and Matrigel Matrix mixture (1:1) and injected subcutaneously into the flank of 4–6 weeks old male nude mice.

### 4.10 Orthotopic Liver Implantation

An appropriate number of luciferase‐labelled cells (MHCC97L: 100,000; PLC/PRF/5: 1,000,000) were suspended in 15 µL Matrigel Matrix. The mixture was orthotopically injected into the left lobe of the liver of a 6–8 weeks old male nude mouse under anesthesia. Six weeks post‐injection, the liver tumour and lungs were examined by bioluminescence imaging using the IVIS Spectrum *in vivo* imaging system (Perkin Elmer). At the end point, mice were sacrificed. Liver tumours and lungs were collected for further analysis.

### 4.11 Sorafenib and Anti‐NID1 mAb Treatment in HCC Mouse Models

Sorafenib powder (Sigma‐Aldrich) was prepared at a working concentration of 200 mg/ml in DMSO. Each mouse was then treated at a concentration of 30 mg/kg in PBS via oral gavage for 21 days consecutively. For anti‐NID1 mAb (DiagnoVEX Therapeutics Limited), each mouse was administered with 400 µg antibody in PBS intraperitoneally, twice a week for 3 weeks.

## Author Contributions

**Cherlie Lot Sum Yeung**: methodology, investigation, data curation, formal analysis, writing – original draft. **Le Cui**: investigation, formal analysis. **Jingyi Wang**: formal analysis, investigation. **Ching‐Ping Chan**: investigation, formal analysis. **Tung Him Ng**: investigation. **Charlotte Jiaqi Lai**: investigation. **Xiaowen Mao**: methodology. **Sze Keong Tey**: methodology. **Michael Shing Yan Huen**: investigation. **Judy Wai Ping Yam**: conceptualization, project administration, supervision, funding acquisition, writing – review and editing, formal analysis.

## Funding

This work was funded by the Research Grants Council General Research Fund (Project No. 17119120) and Theme‐Based Research Scheme (T12‐716/22‐R), Innovation and Technology Commission Innovative and Technology Support Programme Seed Projects (Project No.: ITS/228/20), University Research Committee Seed Fund for Basic Research (Project No. 201910159008 and 202011159039) and Seed Fund for Translational and Applied Research (Project No. 201910160003) of The University of Hong Kong, the Young Researcher Support Scheme of State Key Laboratory of Liver Research (The University of Hong Kong) [Project no. SKLLR/RPG/2021‐g and SKLLR/RPG/2022‐a), and funding support from “Laboratory for Synthetic Chemistry and Chemical Biology” under the Health@InnoHK Program launched by Innovation and Technology Commission, the Government of Hong Kong Special Administrative Region of the People's Republic of China.

## Conflicts of Interest

Judy Wai Ping Yam is founder of DiagnoVEX Therapeutics Limited. Other authors declare no conflict of interest.

## Supporting information




**Supporting Information**: jev270359‐sup‐0001‐SuppMat.pdf

## Data Availability

The data generated in this study are available upon request from the corresponding author.
