# Supplementary material for: Small Extracellular Vesicle‐Derived Nidogen 1 Promotes HCC Carcinogenesis via Transglutaminase 3‐Mediated YAP/TAZ Signalling
Source: J Extracell Vesicles. 2026 Aug 17;15(8):e70359. doi: 10.1002/jev2.70359 (PMC13479469; doi:10.1002/jev2.70359)
Supplement: Supplementary file 1 [file JEV2-15-e70359-s001.pdf]

Stiff matrix induces small extracellular vesicle-derived nidogen 1 promotes HCC carcinogenesis via transglutaminase 3-mediated YAP/TAZ signaling

Cherlie Lot Sum Yeung[1,#], Le Cui[1,#], Jingyi Wang[1,#], Ching-Ping Chan[1], Tung Him Ng[1], Charlotte Jiaqi Lai[1], Xiaowen Mao[2], Sze Keong Tey[3], Michael Shing Yan Huen[4], Judy Wai Ping Yam[1,5,6,*]

[1]Department of Pathology, Li Ka Shing Faculty of Medicine, The University of Hong Kong, Hong Kong
[2]State Key Laboratory of Mechanism and Quality of Chinese Medicine, Institute of Chinese Medical Sciences, University of Macau, Macao
[3]Department of Surgery, Li Ka Shing Faculty of Medicine, The University of Hong Kong, Hong Kong
[4]School of Biomedical Sciences, Li Ka Shing Faculty of Medicine, The University of Hong Kong, Hong Kong
[5]State Key Laboratory of Liver Research (The University of Hong Kong), Hong Kong
[6]Materials Innovation Institute for Life Sciences and Energy (MILES), HKU-SIRI, Shenzhen, China
#These authors contributed equally

Correspondence
*Judy Wai Ping Yam, Department of Pathology, 7/F, Block T, Queen Mary Hospital, Pokfulam, Hong Kong, China. E-mail: judyyam@pathology.hku.hk; Phone: 852-22552681; Fax: 852-22185212

Supplementary Materials and Methods

Supplementary Figures S1-S10

Supplementary Tables S1-S4

Supplementary Materials and Methods

Establishment of expression constructs and stable clones

Guide RNA oligos targeting NID1 and short hairpin RNA (shRNA) oligos targeting TGM3 were synthesized by Integrated DNA Technologies. To establish NID1-KO plasmids, the sgRNA duplexes were subcloned into lentiCRISPR v2-copGFP-Neo via BamHI and MluI sites. NID1-KO plasmids were then transfected to MHCC97L cells by Lipofectamine™ 2000 Transfection Reagent (Thermo Fisher Scientific). Single clones were then picked according to the GFP signal and successful KO was validated by immunoblotting. For the establishment of TGM3-KD cells, shRNA duplexes were subcloned into MISSION® pLKO.1-puro empty vector (Sigma-Aldrich) via AgeI and EcoRI sites. NID1 FL and mutants overexpressing cells were established by XPack™ EV Protein Engineering Technology (System Biosciences) (CMV-XP-MCS-EF1-Puro). NID1 cDNA ORF clone (Sino Biological) was employed for the amplification of NID1 fragment (Accession No. BC045606.1). NID1 FL and mutants were amplified by PCR using primers listed in Supplementary Table S2. The PCR fragment was then purified, digested and subcloned into CMV-XP-MCS-EF1α-Puro Cloning Lentivector via EcoRI and PstI sites. For the establishment of 293T NID1 stable clones, NID1 FL and N677 mutants were subcloned into pMH-SFB (Addgene) plasmid using Gateway® Technology (Invitrogen). Stable cell lines were then established by Lenti-Pac™ HIV Expression Packaging Kit (Genecopeia) into 293FT cells. Target cells were then infected with lentivirus with polybrene transfection reagent (Sigma-Aldrich). Expressions in stable clones were confirmed by immunoblotting.

In vitro siRNA transfection

Cells were seeded in 6-well plates at a density of approximately 5×10^4 cells per well and cultured until they reached approximately 50% confluence. Transient siRNA transfection was performed using Lipofectamine™ 2000 transfection reagent (Thermo Fisher Scientific) and Opti-MEM™ Reduced Serum Medium (Gibco), according to a modified manufacturer's protocol, with a final siRNA concentration of 50 nM. The siRNA sequences used in this study are listed in Table S4.

Nanoparticle tracking analysis

The diameter and particle concentration of sEV in this study were determined by the ZetaView® TWIN NTA PMX-220 (Particles Metrix GmbH). In brief, sEV was diluted to a concentration at around 0.1 ng/ml using Milli-Q® Type 1 Ultrapure Water and injected into the sample cell. Then, 11 positions were captured and analyzed under standard instrumental setting (Laser: 488; Sensitivity: 75; Shutter: 100; temperature: 25℃). Peak analysis and particle concentration were performed by the ZetaView software (version 8.05.11).

Transmission Electron Microscopy (TEM)

Negative staining of sEV

Fresh sEV sample was first diluted to 0.1 µg/µl and fixed by 2% paraformaldehyde solution. Fixed sample (10 µl) was then applied onto discharged copper electron microscope grids for absorption. After 10 mins, the grid was placed on a filter paper for drying and soaked in 2% uranyl acetate for 1 min. The grid was then washed with Milli-Q® Type 1 Ultrapure Water and dry on a filter paper. Images of sEV were visualized on Tecnai G2 20 Scanning TEM and captured by ORIUS SC600 Model CCD Camera linked to Gatan digital micrograph software.

Immunogold labeling of sEV

Isolated sEV samples (around 10 µl each) were added onto Formvar-carbon coated electron microscope grids and allowed for 20 mins absorption. Afterwards, 1% BSA solution was used to block the grids. Primary antibodies were then incubated for 30 mins, followed by incubation of

gold-conjugated secondary antibodies for 20 mins. The grids were washed with PBS and fixed with 1% glutaraldehyde. Images of sEV were visualized by Philips CM100 TEM.

Migration and invasion assays

Transwell® 8 µm polycarbonate membrane inserts (Falcon®) were employed for both assays. An appropriate number of cells were resuspended in 100 µl serum-free medium and added to the upper chamber, while the lower chambers were supplemented with 10% FBS full culture medium. Hepatocyte growth factor (20 ng/ml) was added to the lower chamber as chemoattractant. Cells were allowed to migrate/invade for 16-18 hrs. Afterwards, migrated/invaded cells were fixed with methanol and stained with crystal violet. Cells were imaged and captured under a microscope. Cells were counted from 4 random fields per insert. For the invasion assay, 100 µl of Matrigel matrix (Corning) were used to pre-coat each insert. All experiments were carried out in triplicate.

Colony formation assay

One thousand cells were seeded in a 6-well plate and allowed to incubate for around 7-14 days. Colonies formed were then fixed by methanol and stained with crystal violet. Colonies formed per view were counted.

Cell culture on soft and stiff matrix

CytoSoft® T-75 Rigidity flasks (Advanced Biomatrix) of 0.5 kPa and 8 kPa were employed to mimic the soft and stiff rigidity in cell culture, respectively. PureCol® Type I collagen (Advanced Matrix) was first diluted in 1:30 ratio using DPBS. The diluted ECM solution (30 ml) was then added to coat each flask for 1 hr at room temperature. After incubation, the ECM solution was removed, and the flask was washed twice with PBS and cells were seeded into the flask.

Co-Immunoprecipitation (co-IP)

Protein lysate was extracted from cells with NETN lysis buffer (150 mM NaCl, 50 mM Tris, 5 mM EDTA), supplemented with 10% PhosSTOP^{TM} Phosphatase Inhibitor Cocktail (Roche) and 10% cOmplete^{TM} Protease Inhibitor Cocktail (Roche), for 30 mins at 4℃. The mixture was then subjected to centrifugation at 13,000 × g for 20 mins at 4℃ and supernatant was collected. For each co-IP sample, 500 µg proteins were used and topped up to 500 µl by NETN. Primary antibody (1 µg) was added and allowed for incubation by rotating overnight at 4℃. Protein G Sepharouse^{TM}, Fast Flow (Millipore) beads were prepared by washing with equal volume of NETN for 5 times. Then, 30 µl 50% beads were added to each sample and allowed for incubation by rotating for 4 hrs at 4℃. The beads were then washed with 500 µl NETN for 3 times and 15 µl 2× SDS loading dye was added to each sample. The samples were then boiled for 5 mins at 95℃ and ready for further usage.

Co-IP coupled mass spectrometry (MS) proteomic analysis

Boiled co-IP samples were added to 7.5% polyacrylamide gel and gel electrophoresis was performed. Once the samples reached the boundary between the stacking and resolving gel, electric current was stopped, and the gel was stained by Coomassie Brilliant Blue solution. After being washed by destaining solution (10% acetic acid, 50% methanol and 40% water), the gel containing the sample bands was excised. Sample process, liquid chromatography-MS (LC-MS/MS) and bioinformatic analysis were performed by the Bioinformatics Core, Centre for PanorOmic Sciences (CPOS), LKS Faculty of Medicine, The University of Hong Kong. In brief, in-gel digestion was performed using 10 mM TCEP and 55 mM 2-chloroacetamide for reduction and alkylation. After digestion by trypsin (1 ng/µl) overnight, peptides were extracted from the gel with 50% ACN/5% FA and 100% ACN sequentially. Speedvac-dried peptides were then desalted using C18 StageTips. Eluted peptides were analyzed with Bruker timsTOF pro mass spectrometer using the following

settings (m/z range of 100 to 1700, MS/MS range of 100 to 1700). Raw MS data were processed using MaxQuant 1.6.14.0 and searched against Human Swissprot FASTA database.

RNA sequencing

Total RNA was extracted from cell pellet using ambion® TRIzol™ reagent (Life Technologies) according to manufacturer's instructions. The concentration and purity (OD260/OD280) were quantified using BioDrop spectrophotometer (Biochrom). The cDNA library preparation, Illumina sequencing and bioinformatics analysis were performed by the Bioinformatics Core, Centre for PanorOmic Sciences (CPOS), LKS Faculty of Medicine, The University of Hong Kong. In brief, mRNA containing Poly-A tails were isolated by poly-T oligo-attached magnetic beads and incubated with magnesium ions at 94℃ for 6 mins to create 200-300 bps fragments. The adaptor-ligated libraries were formed by index adaptor ligation (xGen® Dual Index UMI) and 3'adenylation, followed by 15 polymerase chain reaction (PCR) cycles to enrich the ligated libraries. Pair-end 151 bp were sequenced using Illumia sequencer model NovaSeq 6000. The filtered high QC raw reads were mapped (Human Genome GRCh38) and aligned (STAR Version 2.7.8).

Subcellular fractionation for cytoplasmic and nuclear protein extraction

Cytoplasmic and nuclear protein extracts were prepared from HLE and PLC/PRF/5 cells using a commercial subcellular fractionation kit (Thermo Scientific™). Cell pellets were resuspended in ice-cold Cytoplasmic Extraction Reagent I (CER I) according to the packed cell volume, vortexed vigorously for 15 seconds, and incubated on ice for 10 mins. Ice-cold Cytoplasmic Extraction Reagent II (CER II) was then added, followed by a 5-s vortex and a 1-min incubation on ice. After an additional 5-s vortex, the mixture was centrifuged at ~16,000 × g for 5 mins at 4°C. The supernatant containing cytoplasmic proteins was transferred to a pre-chilled tube and kept on ice. The remaining nuclear pellet was resuspended in ice-cold Nuclear Extraction Reagent (NER), vortexed for 15 s, and incubated on ice for 40 mins with intermittent 15-s vortexing every 10 mins. The mixture was then centrifuged at ~16,000 × g for 10 mins at 4°C, and the supernatant containing nuclear proteins was collected in a new pre-chilled tube. Both cytoplasmic and nuclear extracts were quantified using a BCA protein assay, denatured, and subsequently analyzed by immunoblotting.

Immunoblotting analysis

Protein lysates were extracted by mixing cells or sEV with RIPA Lysis and Extraction Buffer (Thermo Scientific™), supplemented with 10% PhosSTOP™ Phosphatase Inhibitor Cocktail (Roche) and 10% cOmplete™ Protease Inhibitor Cocktail (Roche). The mixture was then placed on ice for 30 mins and subjected to centrifugation at 13,300 × g for 20 mins at 4℃. Supernatant was then isolated, and protein concentration was determined by Bradford protein assay. Protein samples (30 µg) were then mixed with 6× SDS sample buffer and heated at 95℃ for 5 mins. Samples were then resolved by SDS-polyacrylamide gel electrophoresis and transferred onto a polyvinylidene difluoride (PVDF) membrane (GE Healthcare), followed by a 1 hr blocking step by 5% non-fat milk or BSA solution. Primary antibodies were then added to the membrane and incubated overnight at 4℃. Afterward, membrane was washed with TBST at room temperature for 10 mins for 3 times and incubated with appropriate horseradish peroxidase (HRP)-conjugated secondary antibodies at RT for 1 hr. The membrane was washed again with TBST at room temperature for 10 mins for 3 times. Chemiluminescence signal was then developed by WesternBright Sirius Chemiluminescent Detection Kit (Advansta Inc) and visualized under Amersham Imager 680 (GE Healthcare).

Cycloheximide treatment

PLC/PRF/5 cells were seeded at a density of 50,000 cells per well in 6-well plates on day 0. After attachment, cells were washed with PBS and treated with sEVs (10 μg/mL) derived from either KO-CTL or KO-NID1 cells for 72 h. Following sEV treatment, cells were incubated in full medium supplemented with 10 μg/mL cycloheximide (CHX) for 0, 3, or 6 hr. At the indicated time points, cells were harvested, and cellular proteins were extracted for western blot analysis.

Enzyme-linked Immunosorbent assay (ELISA)
The level of murine serum AFP was measured by mouse AFP ELISA kit (Meimian), while the circulating sEV-NID1 level in nude mice implanted with PDX subcutaneous tumor was measured by Human Nidogen 1/Enactin (NID1) ELISA Kit (Abbexa), following the manufacturer's instructions. Colorimetric signals were measured at 450 nm using Infinite F200 microplate reader (Tecan).

Isolation of the homemade monoclonal anti-NID1 antibody (anti-NID1 mAb)
To isolate anti-NID1 mAb, hybridoma cells (DiagnoVEX Therapeutics Limited) were cultured until near confluency. Conditioned culture medium was obtained and centrifuged at 1,500 × g for 5 mins to remove dead cells. The supernatant was then extracted and corrected to pH 7.0 by binding buffer (20 mM sodium phosphate, pH 7.0). According to manufacturer's instructions, Protein G GraviTrap antibody purification column (Cytiva) was first equilibrated with 10 ml of binding buffer and cell cultured medium (pH 7.0) was applied to the column. Afterwards, the column was washed with 15 ml binding buffer and mAb was eluted with elution buffer (0.1 M glycine-HCl, pH 2.7). Neutralizing buffer (1 M Tris-HCl, pH 9.0) was added to adjust the pH, and the mAb's concentration was quantified by its absorbance at 280 nm.

Quantitative polymerase chain reaction analysis
HLE and PLC/PRF/5 cells were seeded at a density of 50,000 cells per well in 6-well plates on day 0. After attachment, cells were washed with phosphate-buffered saline (PBS) and treated with sEVs (10 μg/mL) derived from either KO-CTL or KO-NID1 cells for 72 hr. Following treatment, cells were harvested and pelleted by centrifugation. Total RNA was then extracted from the cell pellets using Ambion® TRIzol™ reagent (Life Technologies) according to the manufacturer's instructions. The concentration and purity (OD260/OD280 ratio) of the extracted RNA were quantified using a BioDrop spectrophotometer (Biochrom). Complementary DNA (cDNA) was synthesized from 1 μg of total RNA using the HiScript III RT SuperMix (cat# R323, Vazyme). Quantitative real-time PCR (qRT-PCR) was performed on a LightCycler®480 real-time PCR system (Roche Diagnostics, Mannheim, Germany) with ChamQ Universal SYBR qPCR Master Mix (cat# Q711, Vazyme). The thermal cycling protocol consisted of an initial denaturation step at 95 °C for 5 mins, followed by 40 cycles of denaturation at 95 °C for 15 s, annealing at 60 °C for 30 s, and extension at 72°C for 30 s. Relative mRNA expression levels were calculated using the $2^{-\Delta\Delta CT}$ method, with HPRT1 serving as the internal reference gene for normalization. The specific primer sequences for analyzing TGM3 and HPRT1 are listed in Table S4.

Statistical and data analysis
All statistical tests were analyzed using the GraphPad Prism 9 software. Student's t test was performed to compare the means between 2 groups, while two-way analysis of variance was used for the time-course analysis. Data were expressed as mean± standard error of the mean (SEM). Statistical significance was defined by a two-sided *p* value less than 0.05.

Supplementary Figures

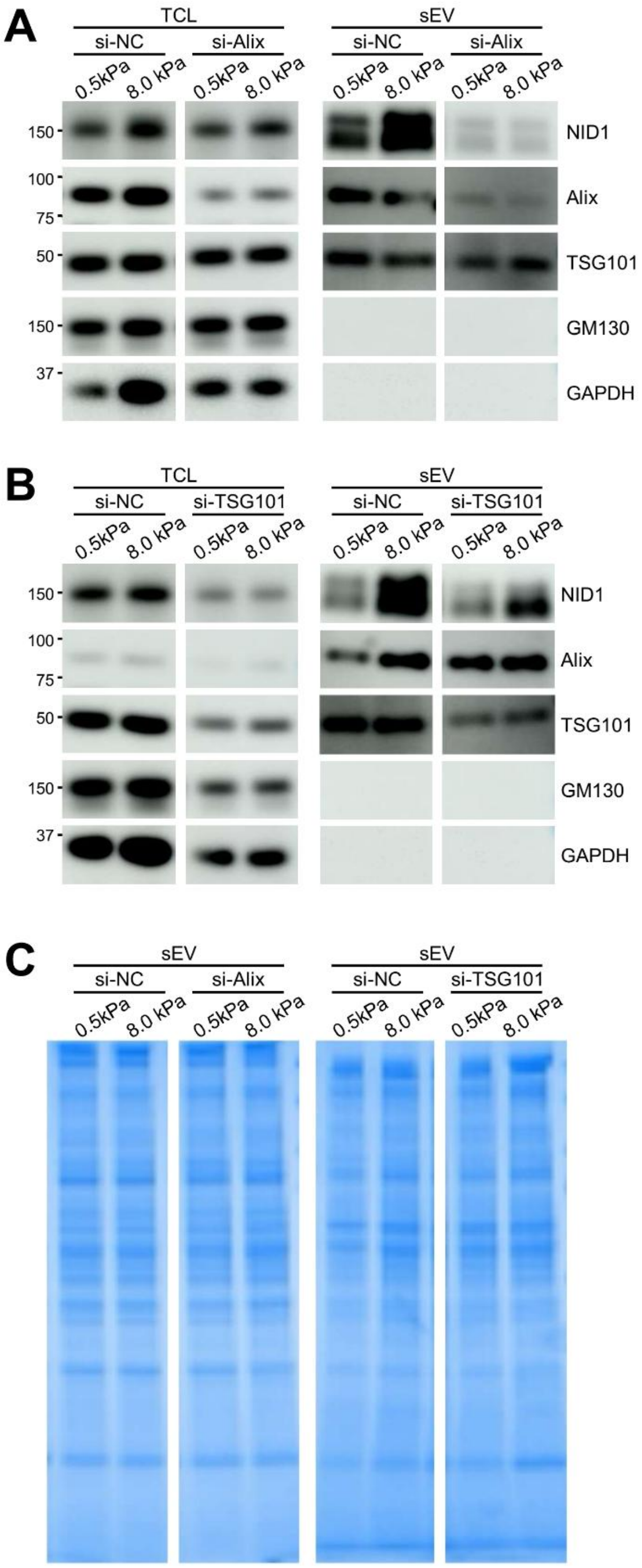

Supplementary Figure S1. Knockdown of Alix or TSG101 reduces NID1 packaging into sEV. HLE cells cultured on soft (0.5 KPa) and hard (8.0 KPa) matrix were transiently transfected with either non-targeting siRNA control (si-NC), siRNA against Alix (si-Alix) (A), or siRNA against TSG101 (si-TSG101) (B). Following transfection, total cell lysate (TCL) and sEV were collected and analyzed by immunoblotting. (C) Image showing Coomassie blue-stained gel fractionated with proteins collected from sEV as loading control.

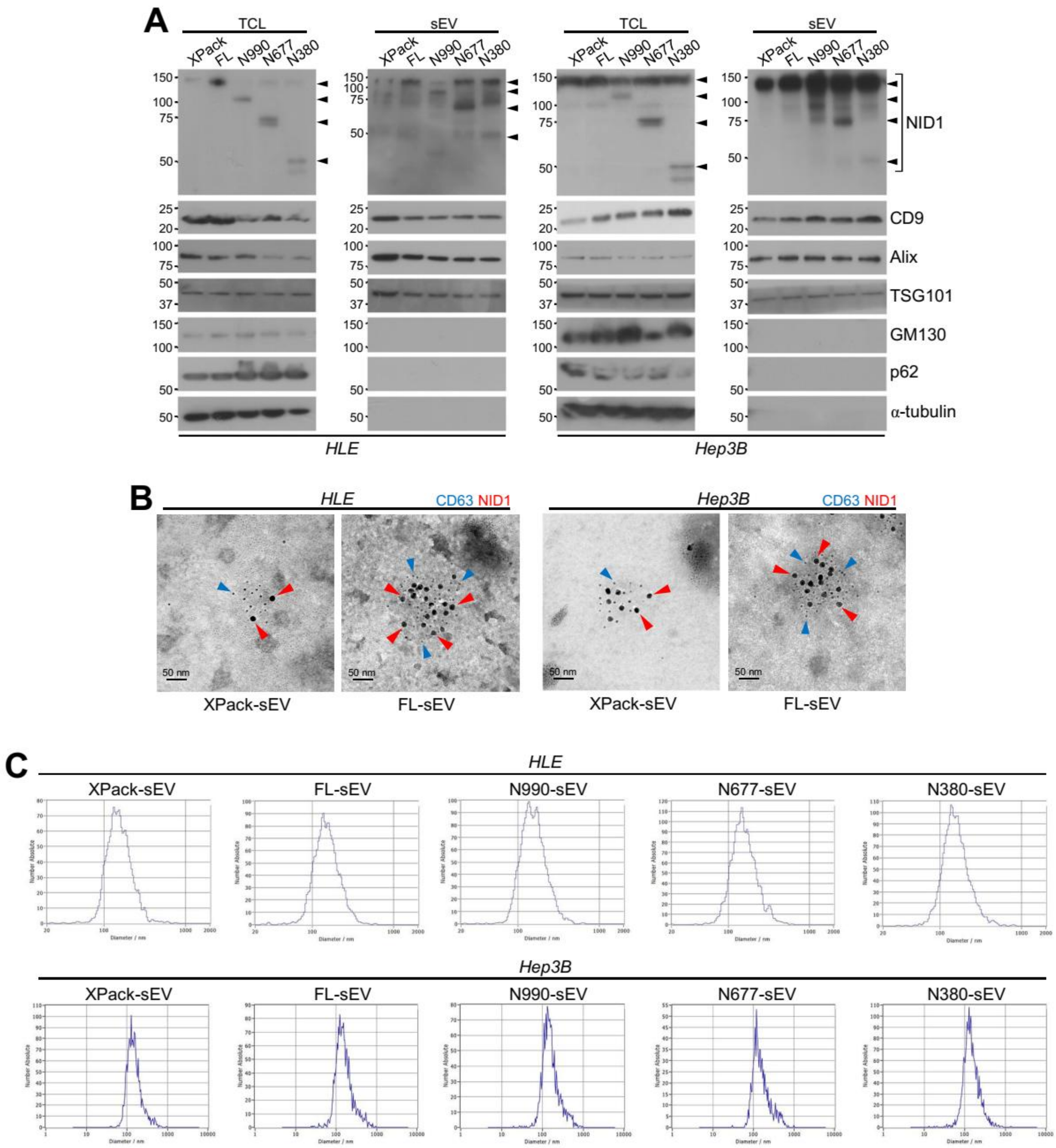

Supplementary Figure S2. Generation and validation of HCC lines expressing NID1 XP FL and mutants.

(A) Immunoblotting of NID1, positive exosome markers (Alix, TSG101 and CD9) and negative exosome markers (GM130, p62 and α-tubulin) in total cell lysate (TCL) and sEV of vector control (XPack), full-length (FL) and mutants (N990, N677 and N380) of NID1 established in HLE and Hep3B cells. (B) Representative electron micrographs of sEV subjected to immunogold labeling using anti-CD63 (blue) and anti-NID1 (red), coupled to 5- and 15-nm gold particles, respectively. Scale bar, 50 nm. (C) The size distribution of sEV derived from these cells was measured by ZetaView® nanoparticle tracking analyzer.

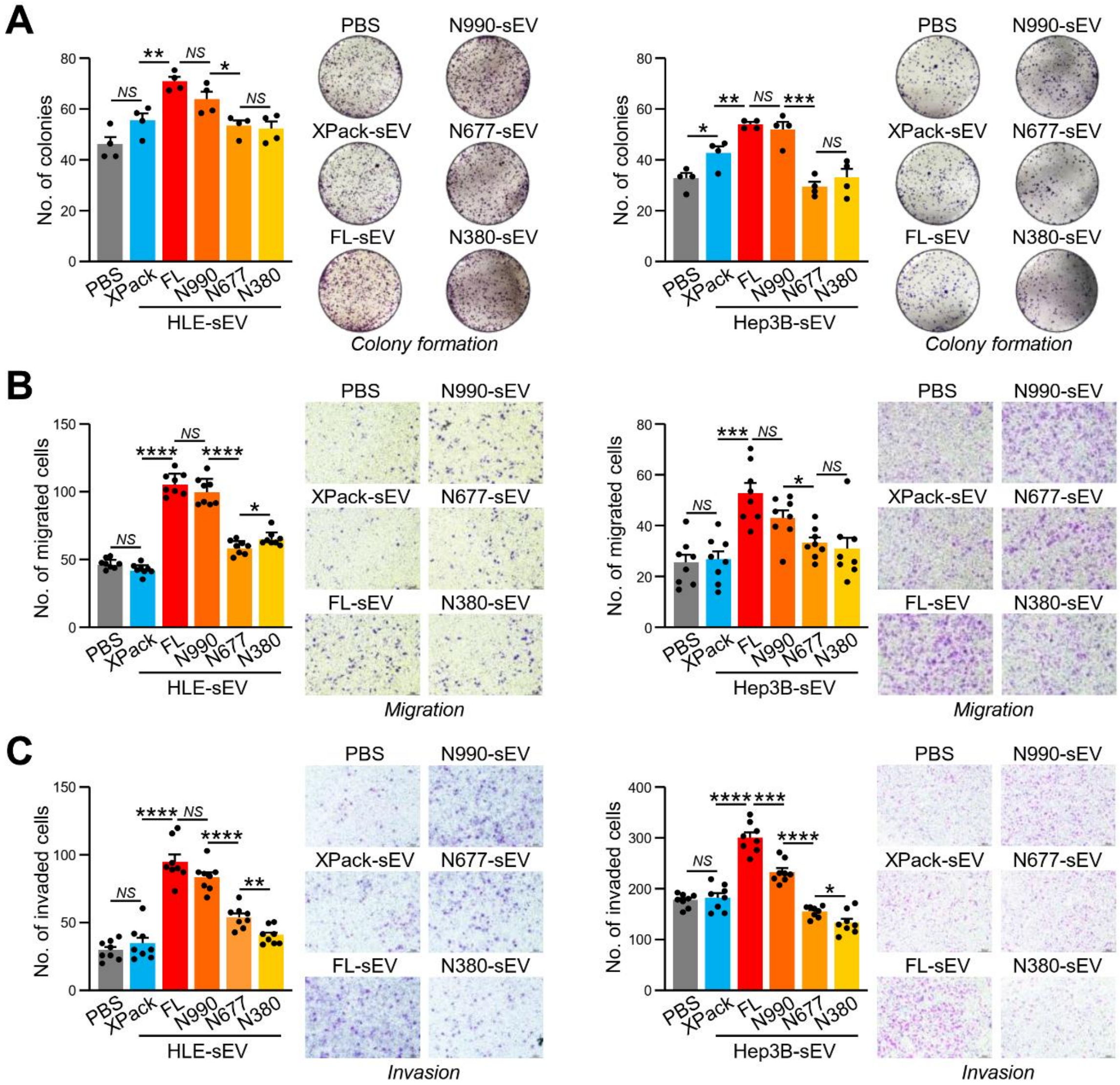

Supplementary Figure S3. Loss of NID1 C-terminus domain diminished sEV-mediated oncogenic activity in vitro.

PLC/PRF/5 cells were treated with PBS or sEV derived from vector control (XPack), full-length (FL) and mutants (N990, N677 and N380) of NID1 established in HLE (*left*) and Hep3B (*right*) cells. Treated cells were subjected to colony formation (A), migration (B) and invasion (C) assays. Representative images and quantification of colonies (A), migrated cells (B), and invaded cells (C) are shown for cells treated with HLE-sEV (*left*) and Hep3B-sEV (*right*).

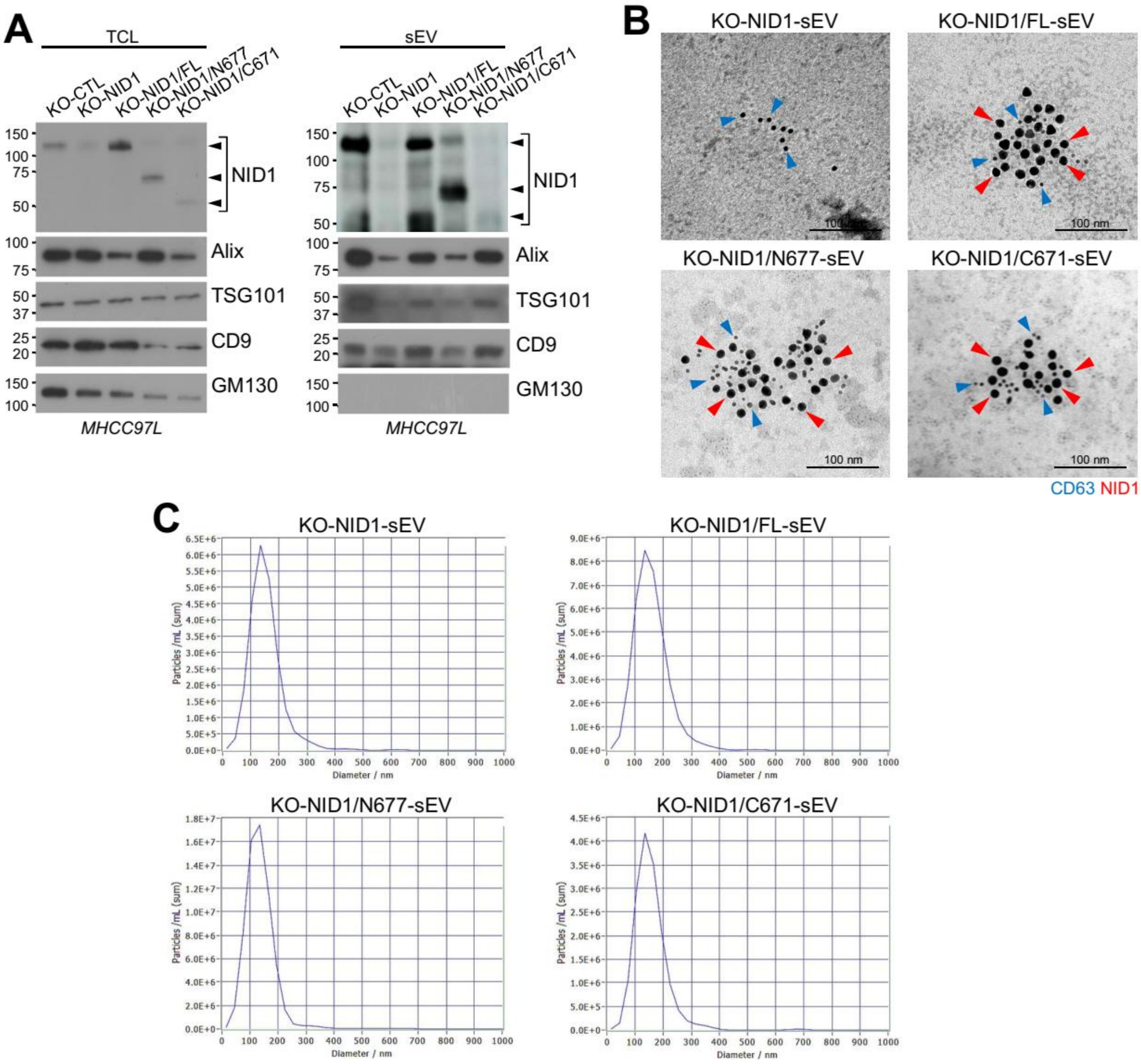

Supplementary Figure S4. Domain-specific NID1 reconstitution (FL/N677/C671) in MHCC97L NID1-KO cells.

(A) Immunoblotting of NID1, positive exosome markers (Alix, TSG101 and CD9) and negative exosome marker (GM130) in total cell lysate (TCL) and sEV from MHCC97L NID1-KO cells overexpressing full-length (FL), truncation mutant (N677) or fragment (C671) of NID1. (B) Representative electron micrographs of sEV subjected to immunogold labeling using anti-CD63 (blue) and anti-NID1 (red), coupled to 5- and 15-nm gold particles, respectively. Scale bar, 100 nm.(C) The size distribution of sEV derived from MHCC97L NID1-KO cells overexpressing FL, truncation mutant (N677) and fragment (C671) of NID1 were measured by ZetaView® nanoparticle tracking analyzer.

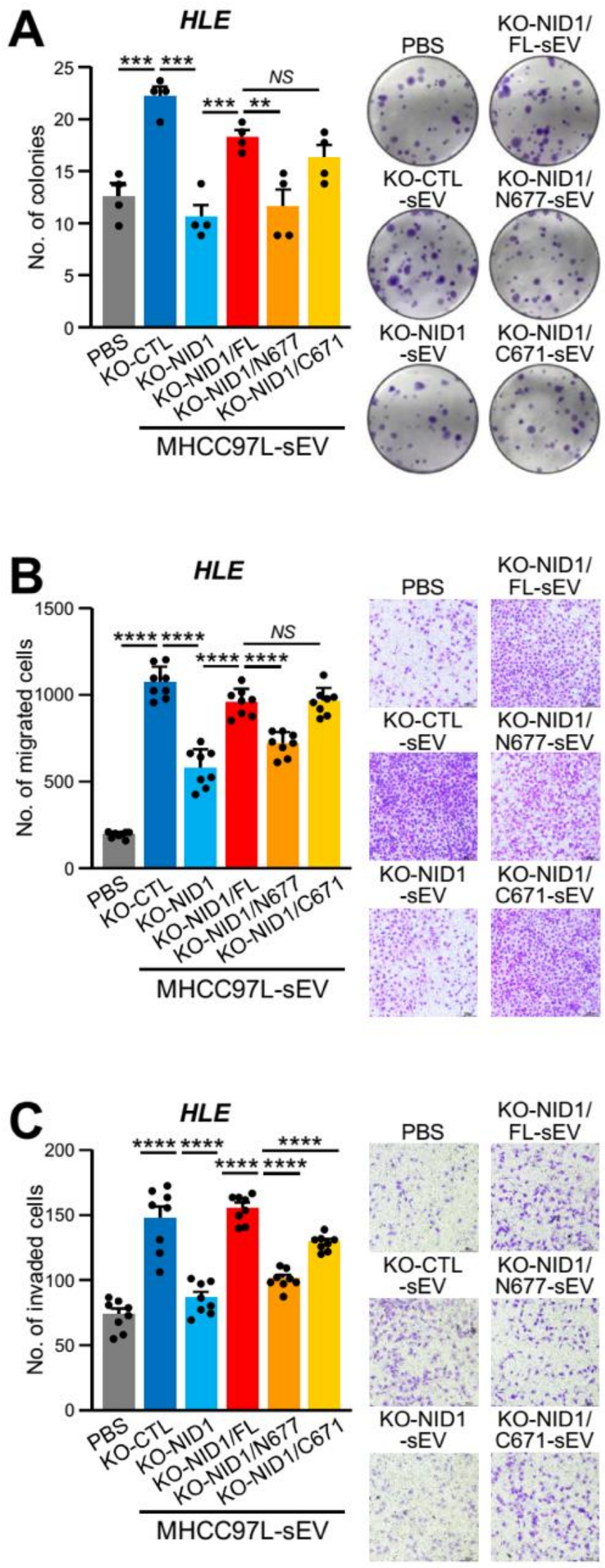

Supplementary Figure S5. C-terminal NID1 rescues sEV-mediated oncogenic phenotypes in vitro.

HLE cells were treated with PBS or sEV derived from MHCC97L control (KO-CTL), NID1-KO, NID1-KO cells overexpressing full-length (FL) and mutants (N677 and C671) of NID1. Treated cells were subjected to colony formation (A), migration (B) and invasion (C) assays. Representative images and quantification of colonies (A), migrated cells (B), and invaded cells (C) are shown.

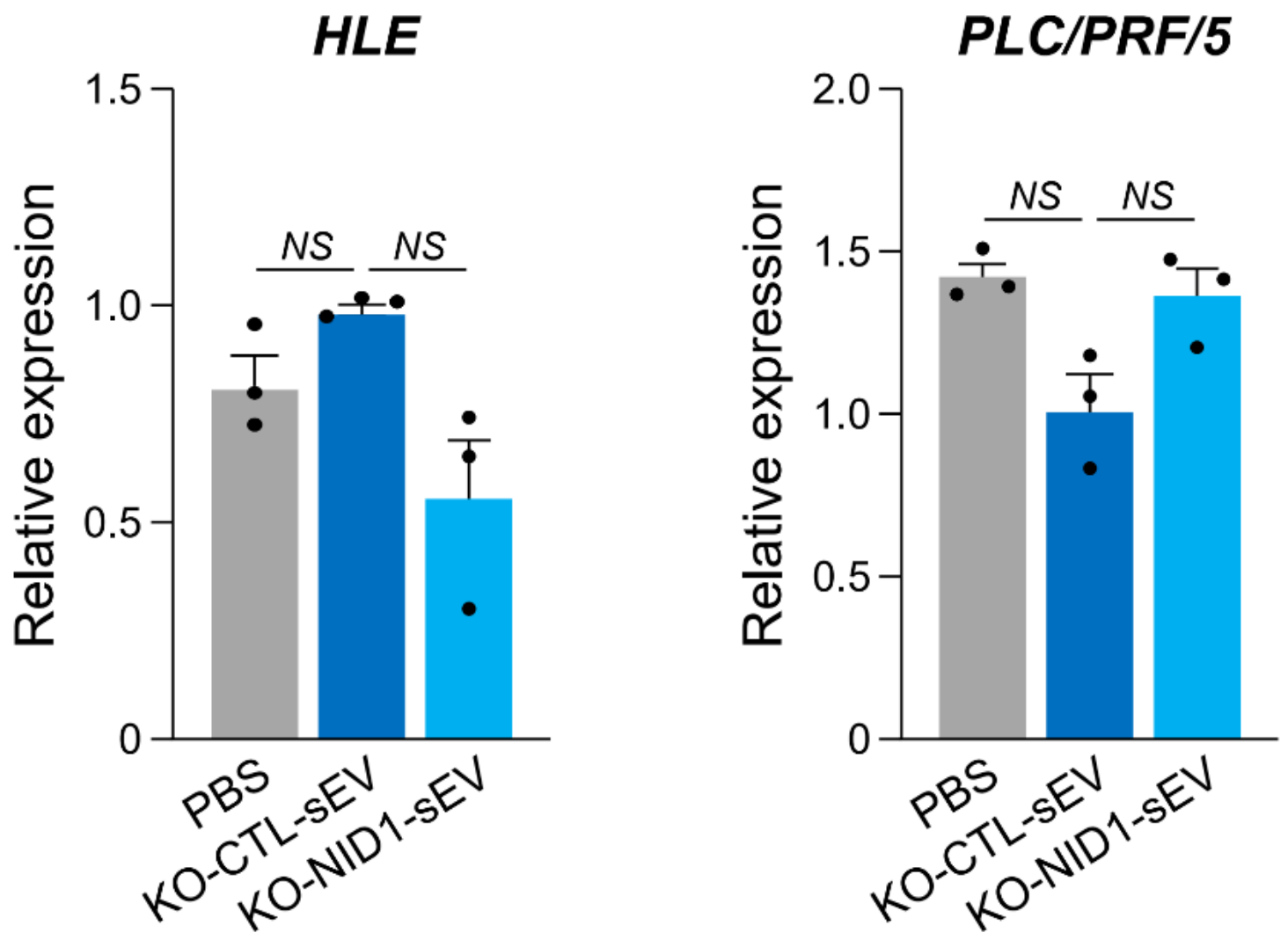

Supplementary Figure S6. sEV-NID1 does not affect transcription level of TGM3 in the recipient cells.
Expression of TGM3 in HLE and PLC/PRF/5 cells treated with or without sEV derived from MHCC97L control (KO-CTL) and NID1 knockout (KO-NID1) cells was analyzed by qPCR.

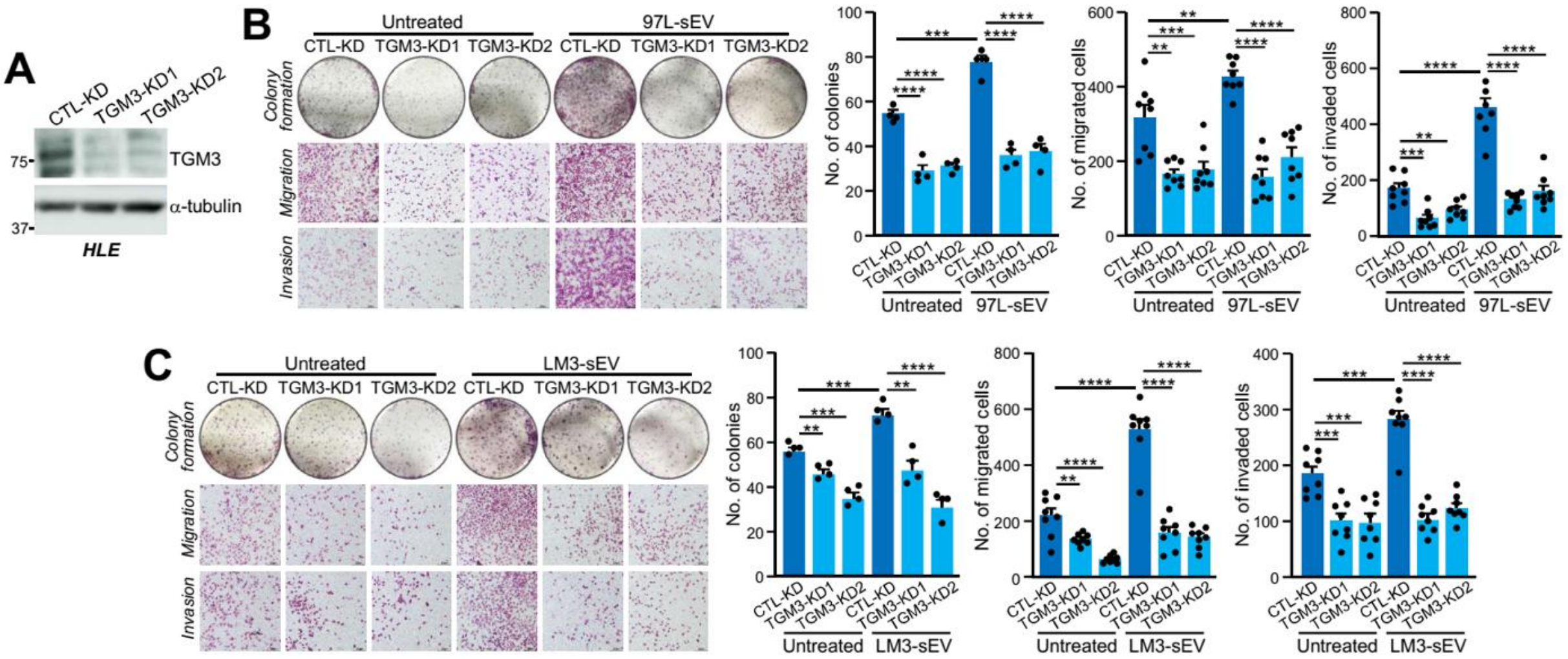

Supplementary Figure S7. TGM3 depletion weakens HCC malignancy and MHCC97L-sEV-driven responses.

(A) Immunoblotting of TGM3 and α-tubulin in CTL-KD, TGM3-KD1 and TGM3-KD2 cells established in HLE. (B-C) HLE CTL-KD, TGM3-KD1 and TGM3-KD2 were treated with PBS, sEV derived from MHCC97L (97L-sEV) (B) and MHCCLM3 (LM3-sEV) (C) cells, respectively. Treated cells were then subjected to colony formation (*left*), migration (*middle*) and invasion (*right*) assays. Representative images and quantification of colonies, migrated cells, and invaded cells are shown.

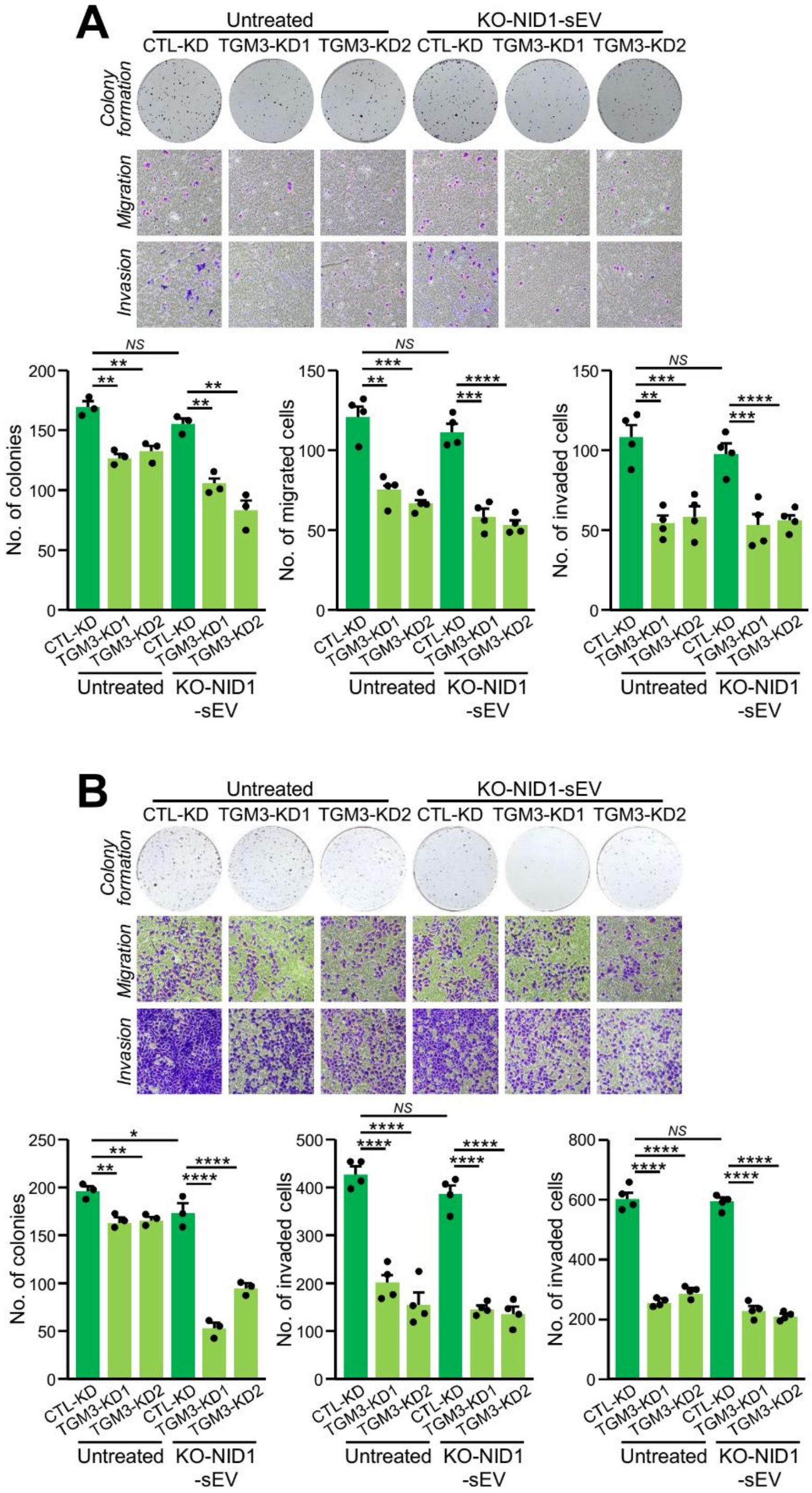

Supplementary Figure S8. TGM3 depletion weakens HCC malignancy and sEV-NID1-driven responses.

CTL-KD, TGM3-KD1 and TGM3-KD2 established in PLC/PRF/5 (A) and HLE (B) were treated with and without sEV derived from MHCC97L NID1 knockout cells (KO-NID1-sEV). The cells were then subjected to colony formation (*left*), migration (*middle*) and invasion (*right*) assays. Representative images and quantification of colonies, migrated cells, and invaded cells are shown.

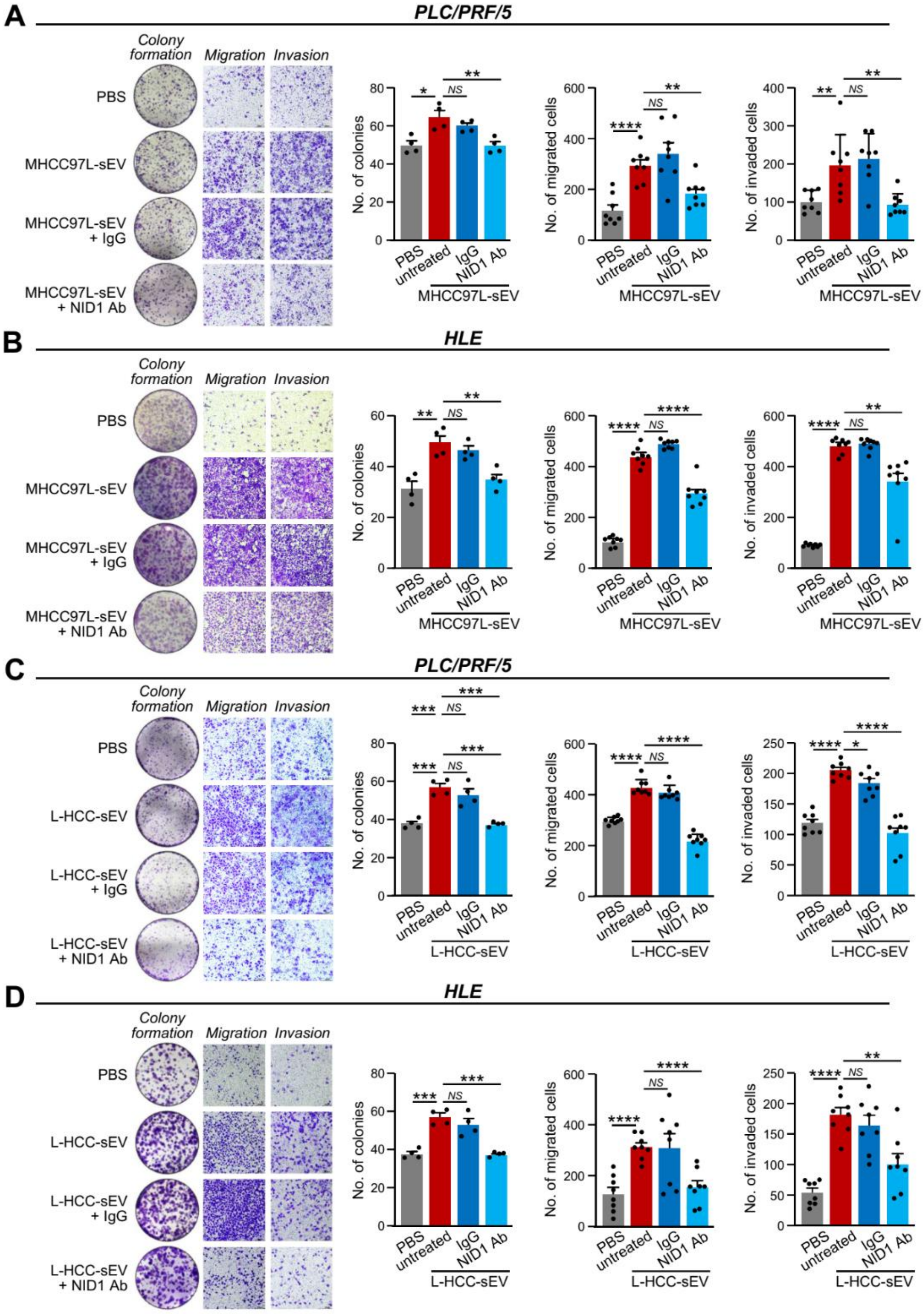

Supplementary Figure S9. Anti-NID1 mAb neutralizes sEV-NID1 from metastatic HCC and patient serum in vitro.

PLC/PRF/5 (A) and HLE (B) cells were treated with PBS and MHCC97L-sEV respectively, with the addition of IgG and anti-NID1 mAb. Treated cells were then subjected to colony formation, migration and invasion assays. Representative images and quantification of colonies, migrated cells, and invaded cells are shown.

PLC/PRF/5 (C) and HLE (D) cells were treated with PBS and sEV derived from late-stage HCC patients (L-HCC-sEV) respectively, with the addition of IgG and anti-NID1 mAb. Treated cells were then subjected to colony formation, migration and invasion assays. Representative images and quantification of colonies, migrated cells, and invaded cells are shown.

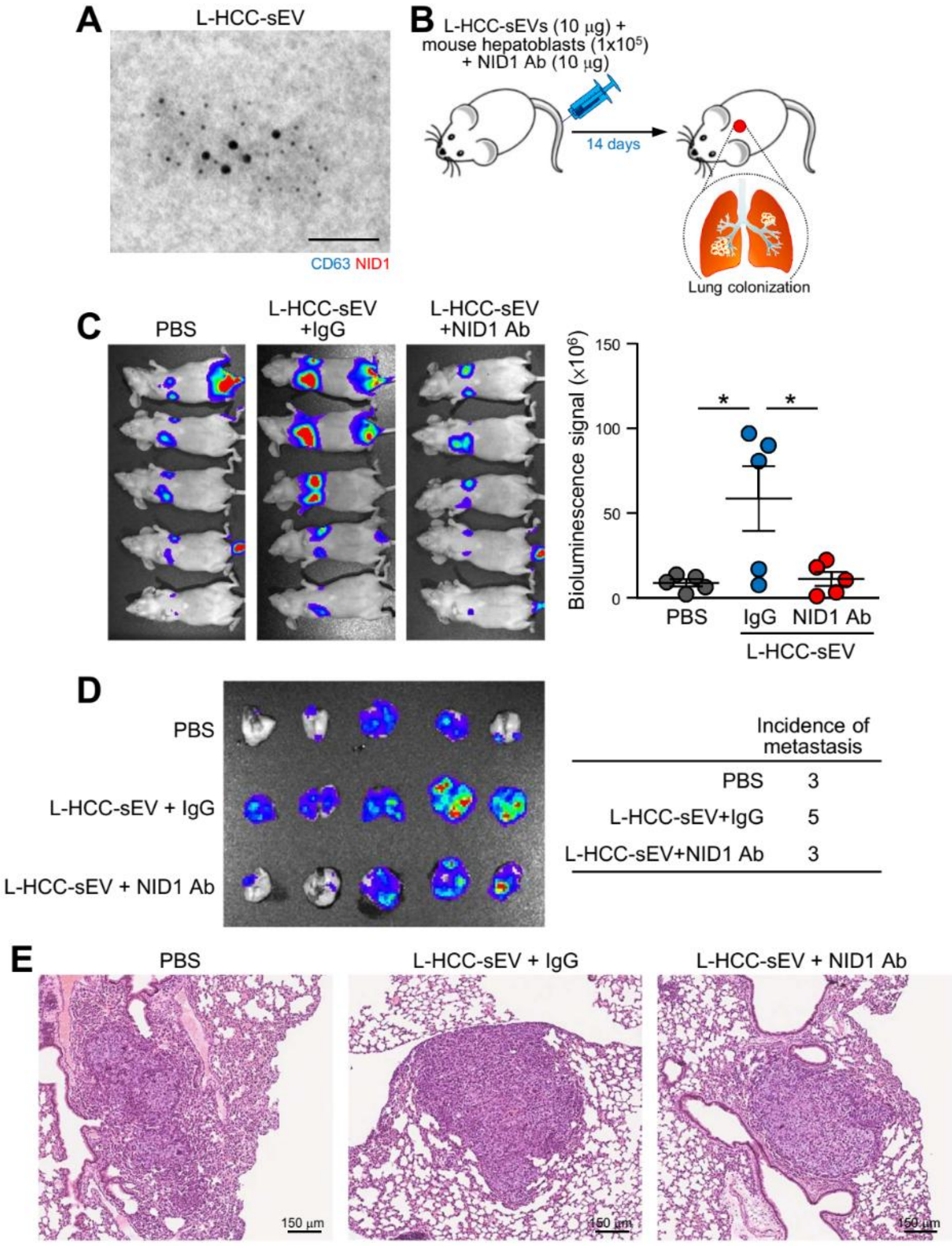

Supplementary Figure S10. Anti-NID1 mAb suppresses L-HCC-sEV-driven lung colonization in vivo.

(A) Representative electron micrographs of sEV derived from late-stage HCC patients (L-HCC-sEV) subjected to immunogold labeling using anti-CD63 (blue) and anti-NID1 (red), coupled to 5- and 15-nm gold particles, respectively. Scale bar, 100 nm. (B) An illustration of lung colonization model in combination with anti-NID1 mAb treatment. Mice were injected with murine p53-/-;Myc hepatoblasts via tail vein, together with PBS or L-HCC-sEV with or without anti-NID1 mAb (10 µg). (C) Bioluminescence signal of whole mouse was captured and quantified. (D) Bioluminescence imaging of excised lung tissues. Incidence of metastasis was recorded. (E) Representative images of H&E-stained lungs and tumor nodules were shown. Scale bar, 150 µm.

Supplementary Table S1. NID1 transcripts detected in HLE and Huh7 cultured in 0.5 and 8 kPa matrix.

HLE

0.5 kPa-HLE -01 normalized read counts	***0.5 kPa-HLE -02 normalized read counts***	***0.5 kPa-HLE -03 normalized read counts***	***8.0 kPa-HLE -01 normalized read counts***	***8.0 kPa-HLE -02 normalized read counts***	***8.0 kPa-HLE -03 normalized read counts***	***Posterior fold change***	***Real fold change***	***Regulation type***	***PPDE***	***FDR***
2012.20	2146.35	2183.86	2676.18	2758.07	2465.00	1.25	1.25	Up	1.00	0.00

Huh7

0.5 kPa- Huh7 -01 normalized read counts	***0.5 kPa- Huh7 -02 normalized read counts***	***0.5 kPa- Huh7 -03 normalized read counts***	***8.0 kPa- Huh7 -01 normalized read counts***	***8.0 kPa- Huh7 -02 normalized***	***8.0 kPa- Huh7 -03 normalized***	***Posterior fold change***	***Real fold change***	***Regulation type***	***PPDE***	***FDR***
16807.27	15993.38	16611.19	17377.02	17230.49	17229.98	1.05	1.05	Up	0.86	0.14

Supplementary Table S2. Binding proteins of NID1 identified by mass spectrometry.

Protein ID	*Protein names*	*Gene names*	*iBAQ[1] SFB-CTL*	*iBAQ SFB-NID1-FL*	*iBAQ SFB-NID1-N677*	*Unique peptides[2] SFB-CTL*	*Unique peptides SFB-NID1-FL*	*Unique peptides SFB-NID1-N677*	*Score[3]*
Q5M775	Cytospin-B	SPECC1	NaN	11.488	NaN	0	4	0	44.575
Q13492	Phosphatidylinositol-binding clathrin assembly protein	PICALM	NaN	11.715	NaN	0	3	0	38.238
A0A494C0J7	Protein-glutamine gamma-glutamyltransferase E	TGM3	NaN	10.175	NaN	0	4	0	27.139
E7EPK0	LIM and calponin homology domains-containing protein 1	LIMCH1	NaN	10.318	NaN	0	4	0	27.030
A0A087X208	Agrin	AGRN	NaN	10.384	NaN	0	4	0	24.956
Q8NBS9	Thioredoxin domain-containing protein 5	TXNDC5	NaN	12.191	NaN	0	3	0	18.539
Q15645	Pachytene checkpoint protein 2 homolog	TRIP13	NaN	10.794	NaN	0	3	0	17.831
Q9BWD1	Acetyl-CoA acetyltransferase, cytosolic	ACAT2	NaN	11.199	NaN	0	2	0	17.768
Q8IWZ3	Ankyrin repeat and KH domain-containing protein 1	ANKHD1	NaN	9.463	NaN	0	2	0	17.197
A2A2D0	Stathmin	STMN1	NaN	13.320	NaN	0	2	0	14.255
E9PMP7	LIM domain only protein 7	LMO7	NaN	10.155	NaN	0	2	0	13.942
O00299	Chloride intracellular channel protein 1	CLIC1	NaN	9.505	NaN	0	2	0	13.170
Q9NQX4	Unconventional myosin-Vc	MYO5C	NaN	7.701	NaN	0	2	0	13.143
O60306	Intron-binding protein aquarius	AQR	NaN	9.466	NaN	0	2	0	13.101
Q9UNF1	Melanoma-associated antigen D2	MAGED2	NaN	9.903	NaN	0	2	0	13.088
Q92665	28S ribosomal protein S31, mitochondrial	MRPS31	NaN	8.689	NaN	0	2	0	13.075
E9PJ21	Hypoxia up-regulated protein 1	HYOU1	NaN	10.467	NaN	0	2	0	12.749
CON__P01966	Hemoglobin subunit alpha	HBA2	NaN	12.381	NaN	0	2	0	12.733
B5MCX3	Septin-2	SEPTIN	NaN	10.529	NaN	0	2	0	12.606
Q5URX0	Beta-hexosaminidase subunit beta	HEXB	NaN	10.536	NaN	0	2	0	11.947
C9JYI4	Importin subunit alpha-5	KPNA1/5/	NaN	11.502	NaN	0	2	0	11.796
A0A0A0MSJ0	ATP-dependent RNA helicase DDX42	DDX42	NaN	9.910	NaN	0	2	0	11.553
O15230	Laminin subunit alpha-5	LAMA5	NaN	6.989	NaN	0	2	0	11.544
P31946	14-3-3 protein beta/alpha; N-terminally processed	YWHAB	NaN	10.552	NaN	0	4	0	11.383
O75489	NADH dehydrogenase iron-sulfur protein 3	NDUFS3	NaN	12.900	NaN	0	2	0	11.175

[1]iBAQ = Log2-transformed, Intensity based absolute quantitation value
[2]Number of unique peptides
[3]The score is correlated with the Q-value

Supplementary Table S3. NID1 and TGM3 docking score.

Receptor interface residues

TYR	818A	4.396	PRO	1227A	3.499
ASN	819A	2.598	GLY	1228A	4.374
THR	820A	2.814	SER	1229A	3.405
PRO	821A	3.098	ARG	1230A	2.648
LYS	829A	4.235	CYS	1232A	4.492
ASP	1072A	2.253	VAL	1241A	3.556
VAL	1074A	4.312	ASP	1242A	3.165
ARG	1075A	2.629			
ASN	1077A	4.483			
TYR	1079A	3.364			
THR	1092A	3.442			
TYR	1094A	2.997			
ASN	1099A	4.678			
ARG	1101A	2.581			
LEU	1103A	3.756			
PHE	1116A	3.845			
ALA	1118A	4.712			
SER	1121A	4.223			
ASN	1137A	2.339			
PRO	1138A	2.473			
SER	1139A	2.633			
GLN	1140A	3.471			
PRO	1141A	3.245			
SER	1142A	3.364			
ARG	1144A	4.578			
HIS	1209A	2.668			
TYR	1211A	2.734			
VAL	1214A	4.885			
ASN	1215A	3.212			
ASN	1216A	3.088			
CYS	1219A	4.774			

Ligand interface residues

THR	60A	4.553
TYR	63A	2.668
TYR	111A	3.405
MET	113A	2.648
LYS	127A	3.165
LEU	128A	3.088
GLY	129A	4.549
PHE	131A	3.815
ILE	188A	3.499
ILE	192A	2.938
ARG	195A	2.734
SER	196A	4.294
ASP	228A	4.396
ASN	230A	4.235
TRP	237A	3.353
GLY	244A	4.323
ARG	245A	2.339
ASN	259A	3.637
TRP	260A	2.633
LYS	261A	4.267
LYS	262A	2.881
SER	263A	2.581
GLY	264A	2.473
PHE	265A	2.253
SER	266A	2.629
PRO	267A	4.455
ARG	269A	3.245
LEU	320A	3.098
LYS	322A	2.598
GLY	323A	2.814
SER	324A	3.863

Receptor-ligand interface residue pairs

818A - 228A	4.396	1101A - 262A	4.662	1209A - 195A	3.647
819A - 237A	3.353	1101A - 263A	2.581	1211A - 195A	2.734
819A - 320A	3.703	1103A - 263A	3.756	1214A - 63A	4.885
819A - 322A	2.598	1116A - 265A	3.845	1215A - 60A	4.553
819A - 323A	4.596	1118A - 265A	4.712	1215A - 63A	4.870
820A - 320A	3.746	1121A - 230A	4.282	1215A - 113A	3.212
820A - 322A	4.697	1121A - 264A	4.854	1216A - 128A	3.088
820A - 323A	2.814	1121A - 265A	4.223	1219A - 128A	4.774
820A - 324A	3.863	1121A - 267A	4.995	1227A - 188A	3.499
821A - 320A	3.098	1137A - 245A	2.339	1227A - 192A	3.499
821A - 323A	4.456	1137A - 264A	4.697	1227A - 262A	3.621
829A - 230A	4.235	1137A - 267A	4.751	1228A - 111A	4.672
1072A - 265A	2.253	1138A - 263A	2.663	1228A - 188A	4.994
1074A - 195A	4.656	1138A - 264A	2.473	1228A - 192A	4.532
1074A - 265A	4.312	1138A - 265A	3.272	1228A - 195A	4.374
1075A - 192A	2.938	1138A - 267A	4.455	1229A - 111A	3.405
1075A - 196A	4.294	1139A - 259A	4.880	1229A - 113A	4.998
1075A - 261A	4.267	1139A - 260A	2.633	1229A - 131A	3.815
1075A - 262A	2.881	1139A - 261A	4.291	1230A - 111A	4.364
1075A - 263A	4.622	1139A - 263A	3.686	1230A - 113A	2.648
1075A - 265A	3.275	1139A - 264A	2.644	1230A - 131A	4.154
1075A - 266A	2.629	1139A - 267A	4.464	1232A - 128A	4.581
1077A - 262A	4.483	1139A - 269A	4.181	1232A - 131A	4.492
1079A - 263A	3.364	1140A - 260A	3.471	1241A - 128A	3.556
1079A - 264A	4.712	1141A - 245A	4.485	1241A - 129A	4.549
1079A - 265A	4.144	1141A - 260A	3.943	1241A - 131A	4.419
1092A - 263A	3.442	1141A - 269A	3.245	1242A - 127A	3.165
1094A - 192A	3.703	1142A - 244A	4.323		
1094A - 262A	2.997	1142A - 245A	3.364		
1099A - 262A	4.678	1144A - 244A	4.578		
1101A - 259A	3.637	1209A - 63A	2.668		

Docking Score : -344.08
Confidence Score: 0.9798
Ligand rmsd (Å): 84.5

Supplementary Table S4. Oligo sequences used in this study.

**Primer name**	**Oligo sequence 5' to 3'**
sg-NID1-F	CGCGTGCCGCAGATTCCGTT
sg-NID1-R	AACGGAATCTGCGGCACGCG
XP-FL-F	GCGAATTCACATGTTGGCCTCGAGCAGCC
XP-C671-F	CGGGATCCCCTGCTACATCGGCACTCA
XP-NID1-Stop-R	AACTGCAGTCATTTCTGTTCGATACAGTCAAC
XP-N990-R	GCGAATTCTCACTTGTCCACGCAGTCAAAGGCCAGT
XP-N677-R	GCGAATTCTCAAGCATCAGGGGAGCCTTCCCT
XP-N380-R	GCGAATTCTCAGAAAACAACTCCTGTTTCCTCAAC
SFB-NID1-FL-F	AACTGCAGTATGTTGGCCTCGAGCAGCC
SFB-NID1-N671-F	AACTGCAGTCCCTGCTACATCGGCACTC
SFB-NID1-Stop-R	AACTGCAGTCATTTCTGTTCGATACAGTCAAC
shTGM3-1F	CCGGGCAATGGCAATACTCTGACTACTCGAGTAGTCAGAG TATTGCCATTGCTTTTTG
shTGM3-1R	AATTCAAAAAGCAATGGCAATACTCTGACTACTCGAGTAG TCAGAGTATTGCCATTGC
shTGM3-2F	CCGGCGTCTTCAATGGGTTTGGAAACTCGAGTTTCCAAACC CATTGAAGACGTTTTTG
shTGM3-2R	AATTCAAAAACGTCTTCAATGGGTTTGGAAACTCGAGTTTC CAAACCCATTGAAGACG
si-Alix-F	GGUGAAGGUCAACGACAAA
si-Alix-R	UUUGUCGUUGACCUUCACC
si-TSG101-F	CCUACUAGUUCAAUGACUA
si-TSG101-R	UAGUCAUUGAACUAGUAGG
TGM3-F	TGGCATGATTGGCTGGAACT
TGM3-R	CCAGCAAGCACACCATTGTC
HPRT1-F	CCTGGCGTCGTGATTAGTGAT
HPRT1-R	AGACGTTCAGTCCTGTCCATAA

Nucleotides in red: start codon, stop codon or residue designated in the deletion mutants
